# Soil Environments Influence Gut Prokaryotic Communities in the Larvae of the Invasive Japanese Beetle *Popillia japonica* Newman

**DOI:** 10.3389/fmicb.2022.854513

**Published:** 2022-04-27

**Authors:** Helena Avila-Arias, Michael E. Scharf, Ronald F. Turco, Douglas S. Richmond

**Affiliations:** ^1^Soil Insect Ecology Laboratory, Department of Entomology, Purdue University, West Lafayette, IN, United States; ^2^Entomology and Nematology Department, University of Florida, Gainesville, FL, United States; ^3^Department of Agronomy, Purdue University, West Lafayette, IN, United States

**Keywords:** hindgut, core microbiota, midgut, transient microbiota, neonate, third instar, 16S rRNA gene survey

## Abstract

Invasive scarab beetles, like the Japanese beetle *Popillia japonica* Newman (JB), spend most of their lives as larvae feeding in the soil matrix. Despite the potential importance of the larval gut microbial community in driving the behavior, physiology, and nutritional ecology of this invasive insect, the role of soil biological and physicochemical characteristics in shaping this community are relatively unknown. Our objectives were to (1) characterize the degree to which larval gut microbial communities are environmentally acquired, (2) examine the combined effects of the gut region (i.e., midgut, hindgut) and local soil environments on gut microbial communities, and (3) search for soil physicochemical correlates that could be useful in future studies aimed at characterizing gut microbial community variation in soil-dwelling scarabs. Gut communities from neonates that were never in contact with the soil were different from gut communities of third instar larvae collected from the field, with neonate gut communities being significantly less rich and diverse. The influence of compartment (soil, midgut, or hindgut) on prokaryotic α- and β-diversity varied with location, suggesting that JB larval gut communities are at least partially shaped by the local environment even though the influence of compartment was more pronounced. Midgut microbiota contained transient communities that varied with the surrounding soil environment whereas hindgut microbiota was more conserved. Prokaryotic communities in the hindgut clustered separately from those of soil and midgut, which displayed greater interspersion in ordination space. Soil cation exchange capacity, organic matter, water holding capacity, and texture were moderately correlated (≥29%) with gut prokaryotic microbial composition, especially within the midgut. Findings suggest that microbial communities associated with the JB gut are partially a function of adaptation to local soil environments. However, conditions within each gut compartment appear to shape those communities in transit through the alimentary canal.

## Introduction

A diverse variety of beetles inhabit the soil, and many of them feeds on lignocellulose-rich plant material in different stages of decay, from living plant roots to humus (Smith, 1922; Raw, 1967; Jackson and Klein, 2006; Zhang and Jackson, 2008; Huang et al., 2010; Brune, 2019). To enable exploitation of available food sources, the larvae of scarab beetles (family Scarabaeidae) often possess a strongly alkaline midgut equipped with alkali-stable hydrolytic enzymes capable of degrading lignocellulose into its constituent, fermentable sugars (Crowson, 1981b; Terra, 1990; Lemke et al., 2003; Gupta and Bisaria, 2018; Chouaia et al., 2019). Additionally, the scarab larval hindgut includes an expanded, microbe-rich, anaerobic fermentation chamber (Crowson, 1981b; Terra, 1990) hosting a diverse community of symbiotic microorganisms that facilitate the digestive process (Crowson, 1981b; Cazemier et al., 1997; Lemke et al., 2003; Huang et al., 2010). As with many other arthropods (Cazemier et al., 1997; Douglas, 2015; Brune, 2019; Scharf and Peterson, 2021), the hindgut of scarab larvae is believed to function analogously to the rumen of ungulates, where microbial fermentation is essential for optimal nutrient acquisition (Crowson, 1981a). The forces shaping the composition of these ecologically important gut microbial communities are still coming into focus.

In general, the insect gut microbiota is primarily composed of core and transient microorganisms, either of which may be obtained through vertical (heritable) and horizontal (environmental sourcing) acquisitions (Engel and Moran, 2013; Douglas, 2015). The vertical acquisition could occur either in (transovarial), on (egg smearing of the chorion during oviposition), or near (egg capsule transfer) eggs (Kucuk, 2020; Nyholm, 2020). Additionally, parental care of offspring potentially promotes the vertical transmission of symbionts. In the case of environmental sourcing, appropriate symbiotic microorganisms may be selectively cultivated from a wide range of organisms as they are ingested and passed through the insect gut (Dematheis et al., 2012; Shapira, 2016). Interspecific variation in the composition of insect gut microbial communities appears to be driven by feeding habits, habitat, phylogeny, and gut morphology and physiology (Yun et al., 2014; Kudo et al., 2019; Ebert et al., 2021). In contrast, intraspecific variation in gut microbial communities may be driven by the insect developmental stage (Crowson, 1981d; Hammer and Moran, 2019; Kucuk, 2020), gut compartment (Egert et al., 2003; Lemke et al., 2003; Zhang and Jackson, 2008; Andert et al., 2010; Chouaia et al., 2019), diet (Zhang and Jackson, 2008; Andert et al., 2010; Franzini et al., 2016), and local environmental variables (Pittman et al., 2008; Zhang and Jackson, 2008; Huang and Zhang, 2013; Macke et al., 2017). The influence of local environmental variables, particularly the chemical and biological aspects of the soil substrate, in shaping gut microbial communities remains understudied in soil-dwelling scarab larvae.

Soil-dwelling scarab larvae continuously ingest a mixture of inorganic and organic soil components (Crowson, 1981b; McQuillan and Webb, 1994; Millas and Carrillo, 2010), including microorganisms adhered to plant material and soil particles (Gan and Wickings, 2020). Through this constant processing of soil and its highly diverse microbial constituency (Fierer, 2017; Jansson and Hofmockel, 2018), scarab larvae will acquire a mixture of symbiotic and transient (i.e., those passing through the alimentary canal either unchanged or removed through attrition) gut microbiota. Once inside the insect, ingested microbiota is quickly transformed such that communities from the ingested soil matrix only partially resemble those of the alimentary tract (Egert et al., 2003; Andert et al., 2010; Arias-Cordero et al., 2012; Chouaia et al., 2019). Further still, microbial communities in the hindgut, where a diverse assortment of microbial-driven processes often occur (Crowson, 1981b; Cazemier et al., 2003; Lemke et al., 2003; Andert et al., 2010), are richer and more abundant than those of the midgut (Cazemier et al., 1997; Egert et al., 2005; Zhang and Jackson, 2008). Therefore, the gut microbial communities of soil-dwelling scarab larvae could be shaped by host physical and physiological forces, as well as by external soil-related factors.

Relatively few studies have examined the impact of local environmental factors, including soil substrate, on the composition and diversity of gut microbial communities found in soil-dwelling scarab larvae. Zhang and Jackson (2008) found that the bacterial communities in the midgut of larval *Costelytra zealandica* (Scarabaeidae) varied between samples and locations, whereas those in the hindgut contained a more diverse but stable bacterial community that was less affected by external conditions. Similar results have been reported for the larval hindgut of other scarabs, including *Dermolepida albohirtum* (Pittman et al., 2008), *Holotrichia parallela* (Huang and Zhang, 2013), and *Pachnoda* spp. (Andert et al., 2010). However, these previous studies all employed DNA fingerprinting techniques (i.e., PCR-DGGE or TRFLP) which allowed microbial community comparisons but at relatively low resolution compared to more modern techniques (Lynch and Neufeld, 2015; Hugerth and Andersson, 2017). No studies to date have used high-throughput DNA sequencing to interrogate how biotic or abiotic characteristics of the soil substrate may influence gut microbial recruitment in soil-dwelling scarab larvae.

The Japanese beetle (JB), *Popillia japonica* Newman (Coleoptera: Scarabaeidae) is an economically important insect (United States Department of Agriculture, 2015) that spends most of its life in the soil as larvae, feeding on roots of various plants and soil organic matter (Smith, 1922; Britton and Johnson, 1938). Given its invasiveness, growing global distribution (Kistner-Thomas, 2019), and apparent ability to quickly adapt to new environments, some plasticity in the composition of the larval gut microbial community may be ecologically advantageous (Chu et al., 2013; Macke et al., 2017; Xu et al., 2019). In the single published study addressing gut microbial diversity in JB, researchers concluded that soil microbes represent an essential source of larval gut bacteria and that many of the microbial taxa present were potentially involved in the degradation of ingested plant material (Chouaia et al., 2019). Two key unanswered questions are (1) the degree to which JB gut microbiota are environmentally acquired, and (2) whether the gut microbiota displays plasticity in response to variation in host soil. To answer these questions, we first compared the communities of neonate larvae eclosing from surface-sterilized eggs to those of third instar larvae collected from the field using two geographically disparate populations. We then examined the combined effects of the gut region (i.e., midgut, hindgut) and local soil environments in shaping JB larval gut microbial communities using high throughput Illumina sequencing. In light of the invasiveness of this species, we also searched for correlations between soil biological and geochemical characteristics, and gut microbial communities that could be useful in future studies. Our findings provide a clearer picture of the forces driving gut microbial communities in JB, as well as the microbial constituents that may be considered core microbiota vs. those that are simply generalist or transient residents of the alimentary canal.

## Materials and Methods

### Environmental Sourcing of JB Prokaryotic Gut Microbiota

To characterize the degree to which JB larval gut microbiota may be environmentally sourced, we compared the prokaryotic communities of whole gut homogenates taken from third instar larvae to those of neonates freshly eclosed from surface-sterilized eggs.

Neonates were obtained from two spatially distant (>320 km) populations by collecting adults in Indiana (Throckmorton-Purdue Agricultural Center, TPAC) and Wisconsin (Janesville) (Table 1) and allowing them to oviposit into a uniform soil habitat under laboratory conditions. Adult JB were collected in July 2018 using Trece^®^ catch-can traps (Trece, Inc, Adair, OK, United States) baited with a floral lure. Adults from Janesville were placed into Ziploc bags containing apple wedges as a source of food and moisture and shipped overnight inside a cooler with ice packs to the soil insect ecology laboratory at Purdue University. Adults from TPAC were transported to the lab inside plastic storage boxes the same day they were collected. Adults from Janesville and TPAC were held overnight at 15°C inside plastic boxes containing fresh apple wedges. The following day, 100 adults (50:50 sex ratio) were placed inside 6.8 L plastic storage boxes containing soil from TPAC that had been prepared for oviposition by removing visible plant residues and rocks and passing them through a 2 mm sieve. Each plastic box contained enough soil to achieve a 7 cm depth and three boxes were separately allocated to adults from each population. Apple wedges were provided as a source of food and moisture and a tight-fitting lid, with the center cut out and replaced with a nylon window screen, was immediately placed on each box to contain the beetles. Beetles were allowed to feed, mate, and oviposit for 1 week at room temperature (21°C). During this time, the soil was moistened daily with sterile water using a hand-operated spray bottle, and apple wedges were replaced every 2–3 days.

**Table 1:** Soil properties at locations where Japanese beetle (*Popillia japonica* Newman) larvae were collected for gut microbial community analysis.

				**Soil texture^b^**							
**Location**	**Infestation**	**U.S. State**	**GPS coordinates^a^**	**Sand (%)**	**Silt (%)**	**Clay (%)**	**Classification^c^**	**Organic Matter (%)**	**pH**	**CEC^d^**	**WHC^e^**
Blackhawk	Natural	Wisconsin	43.076500N, −89.463510W	17	66	17	Silt loam	5.6	6.4	12.6	34.2
Culver	Natural	Indiana	41.219323N, −86.395881W	88	10	2	Sand	10.5	6.5	11.1	26.3
Janesville1	Natural	Wisconsin	42.696356N, −89.059291W	22	59	19	Silt loam	8.8	6.8	16.8	37.9
Janesville2	Natural	Wisconsin	42.696112N, −89.056620W	46	40	14	Loam	4.5	6.7	10.8	24.1
Nursery	Artificial	Indiana	40.419712N, −86.940559W	63	31	6	Sandy loam	3.8	5.0	10.5	19.2
Purdy	Natural	Indiana	40.369023N, −86.903741W	22	58	20	Silt loam	3.8	6.0	8.3	27.2
TPAC^f^	Artificial	Indiana	40.295484N, −86.895378W	27	51	22	Silt loam	6.1	7.3	14.3	35.6

a *UTM Zone 16;*

b *Determined by the hydrometer method (Bouyoucos, 1962);*

c *According to the United States Department of Agriculture–Natural Resources Conservation Service (USDA-NRCS);*

d *CEC, Cation Exchange Capacity (meq/100 g);*

e *WHC, Water Holding Capacity at 1/3 Bar;*

f *TPAC, Throckmorton-Purdue Agricultural Center*.

After 1 week, live adults were removed from the containers, and soil was sieved (2 mm) to recover the eggs. Eggs were cleaned with a fine paintbrush to remove soil residue, submerged in sterile distilled water for 2 min, and rinsed three additional times using a low-pressure stream of sterile distilled water. Eggs were then surface sterilized by immersing them in a 10% sodium hypochlorite solution for 2 min, followed by a triple rinse with sterile distilled water. Using a sterile technique, surface-sterilized eggs were then transferred into sterile Petri dishes fitted with moist filter paper and incubated at room temperature (21°C). Eggs were inspected daily until eclosion. Once eclosed, neonates were transferred into sterile Petri dishes, and their alimentary tracts dissected aseptically under a stereomicroscope (Supplementary Figure 1A). Alimentary tracts were placed in DNA buffer tubes and store at −20°C until processed. To secure enough gut material for DNA extraction, the dissected alimentary tracts of 10 neonates were pooled together to form one replicate. Samples from third instar larvae were collected from Janesville and TPAC as described below.

### Variation in the Microbiota of Third Instar Larval Guts and Soil

#### Field Locations

To characterize variation in the prokaryotic microbiota of third instar JB larval guts and infested soil, samples were collected from seven locations across Indiana and Wisconsin, USA (Table 1). Moreover, five locations consisted of natural infestations occurring under monocultures of Kentucky bluegrass maintained as turfgrass. In one location (TPAC), the infestation was created artificially on agricultural soil subjected to 30+ years of rotational corn and soybean. TPAC thus provided an opportunity to compare gut microbial communities in JB larvae from soils with much different management histories. An additional, manipulated larval treatment (Nursery) was specifically designed to examine how gut microbiota of third instar larvae collected from a given location could be altered by incubating those larvae in soil from an alternate location.

##### Naturally Infested Locations

At each location, third instar larvae and soil samples were collected the same day. Third instar larvae were collected using a soil coring device to remove a 10.8 cm diameter core of soil and verdure to a depth of ~10 cm. Soil and root material were broken apart by hand, and all scarab larvae were collected and placed into plastic Ziplock bags containing soil from that location. These samples containing larvae were labeled, placed into a cooler with ice packs, transported to the laboratory, and stored in a low-temperature incubator at 15°C until dissection. Composite soil samples for the microbial survey consisted of 20 soil cores (1 cm diameter × 2.5 cm depth) collected from each location. These samples were placed into a labeled plastic Ziplock bag and transported to the laboratory in a cooler with ice packs. Composite soil samples were sieved (2 mm) after removing thatch, visible plant residues, stones, and all discernable invertebrates, and stored at 4°C. The soil was then weighed and placed in a DNA extraction buffer at −20°C until processed. Soil texture (sand:silt:clay), organic matter content, pH, and cation exchange capacity (CEC) were determined by A&L Great Lakes (Fort Wayne, IN, USA) following their standard procedures (https://algreatlakes.com).

##### Artificially Infested Location

The artificial infestation at TPAC was created by driving a total of seventy-two PVC cylinders (20.3 cm diameter ×15 cm deep) into the soil to a depth of 10 cm so that 5 cm of the cylinder remained above the soil surface. In May 2018, the surface of the soil within each cylinder was lightly scarificed using a hand rake, and a 20 cm diameter disk of Kentucky bluegrass sod (2.5 cm of soil) was placed inside each. The sod was immediately irrigated by filling the remaining depth of each cylinder with water, and the irrigation process was repeated as necessary to minimize stress and promote the establishment of the sod. The cylinders at TPAC were infested during July 2018, by caging adult JB that were collected locally. These adults were collected using Trece^®^ catch-can traps baited with a floral lure, returned to the laboratory, and held overnight at 15°C inside plastic storage boxes. Apple wedges were provided as a source of food and moisture. The next day, adults were sorted into groups of 40 (50:50 sex ratio) and placed inside 2 oz plastic deli cups containing an apple wedge. Lids were placed on the cups to contain the beetles which were then transported to TPAC inside a cooler with ice packs. On arrival, the contents (beetles and apple wedge) of one plastic deli cup were placed inside each cylinder. To contain the beetles, a nylon window screen was placed over the open end of each cylinder and secured using a plastic snap-top lid (model L808, Berry Global, Evansville, IN, USA) with the center section (16 cm diameter) removed to allow air and water to pass through. Beetles were allowed to feed and oviposit for 1 week and the caging process was repeated using fresh, field-collected beetles. The resulting third instar JB larvae were collected in September 2018, using a shovel to pry the cylinders from the ground. The soil and root material within each cylinder were broken apart to reveal the larvae. Soil and larvae were handled as described for the natural infestations.

##### Manipulated Larval Treatment

To further discern the effect of soil environment on gut microbial communities, a subset of JB larvae collected from the Purdy location was incubated in previously uninfested soil taken from the Purdue Nursery. Larvae were cleaned with a paintbrush to remove all soil remaining from Purdy and transferred to a 1 L glass jar containing 400 g of sieved (2 mm) uninfested soil collected from the Nursery. Larvae were incubated at room temperature (21°C) for 7 days. Since transit time for substrate through the digestive tract of JB larvae has not been reported, we guided the incubation time based on the report for other soil-dwelling root-feeding Scarabaeidae species, which was from 2 days (Millas and Carrillo, 2010) up to ~5 days (4–8 h in the midgut and up to 4 days in the hindgut) (Egert et al., 2005). Again, soil and larvae were handled as described for the natural infestations.

#### Third Instar Larval Gut Dissection

To prepare for gut dissection, third instar larvae were identified to species rank based on the confirmation of the raster pattern (Richmond, 2017), and soil particles were removed from the larvae using a clean paintbrush. Larvae were flash frozen at −20°C for 20 min, submerged in 70% ethanol for 10 min, then rinsed with 70% ethanol and sterile distilled water. Gut contents were aseptically dissected and divided into midgut (between the first set of gastric caeca after the head and the pyloric sphincter where the Malpighian tubules emerge) and hindgut (including ileum, colon, and rectum) sections (Supplementary Figure 1A). Midgut and hindgut sections were placed separately in DNA extraction buffer and stored at −20°C until processed.

### Genomic DNA Extraction and Quantification

Total genomic DNA was extracted from samples (i.e., neonate larval whole guts, third instar larval midguts and hindguts, and soils) using the DNeasy Power Soil Kit (Qiagen, Valencia, CA, USA) following manufacturer instructions. The quality and purity of DNA were assessed using the NanoDrop 2000 UV-Vis Spectrophotometer (Thermo Fisher Scientific Inc., Wilmington, DE, USA), based on the absorbance ratios of 260/280 nm (1.8–2) and 260/230 nm (>1.7). The integrity of the DNA was also confirmed by electrophoresis in a 1% agarose gel with 1 × TAE buffer. DNA concentrations (ng/μL) in samples ranged from 47.9 to 80.6 (62 ± 10.5) for neonates, from 101.4 to 363.1 (228.3 ± 79.5) for soil, from 23.3 to 85.8 (52.2 ± 17.3) for midgut, and from 35.2 to 202.9 (80.7 ± 35.1) for hindgut. Extracted genomic DNA was stored at −20°C before amplification and sequencing.

### 16S rRNA Gene Amplification and Sequencing

Bacterial and archaeal 16S rRNA gene-spanning libraries were generated at the Environmental Sample Preparation and Sequencing Facility (ESPSF) at Argonne National Laboratory (Lemont, IL, USA) following the Earth Microbiome Project benchmarked protocol (http://www.earthmicrobiome.org/emp-standard-protocols). The V4 hypervariable regions of the bacterial and archaeal 16S rRNA gene region were amplified using the primers 515F (5′-GTGYCAGCMGCCGCGGTAA-3′) and 806R (5′-GGACTACNVGGGTWTCTAAT-3′). Pooled amplicons were sequenced on a multiplexed Illumina MiSeq 2 × 151-bp platform at ESPSF.

### Bioinformatics and Statistical Analysis

Bioinformatic analysis was performed using QIIME2 2020.2 (Bolyen et al., 2019). Raw FASTQ reads were demultiplexed and quality filtered using the q2-demux (emp-paired) plugin followed by denoising using DADA2 (Callahan et al., 2016). Further filtering of features was applied to reduce likely sequencing errors; retained features were observed in at least 2 samples, were assigned at least to the taxonomic rank of phylum, and were not classified as mitochondria or chloroplast.

The resulting amplicon sequence variants (ASVs) were aligned with mafft (Katoh et al., 2002) using the q2-alignment plugin and a phylogeny was constructed with fasttree2 (Price et al., 2010) using the q2-phylogeny plugin. ASVs were annotated using q2-feature-classifier (Bokulich et al., 2018) with a taxonomy classify Scikit-learn (Pedregosa et al., 2011) pre-trained to primers using the Silva database (released 132, April 2018) (Quast et al., 2012; Glöckner et al., 2017) at 99% similarity. ASVs with the same taxonomic assignment were collapsed using the q2-taxa collapse plugin. To examine the degree to which gut microbiota in JB larvae are environmentally sourced, gut microbial communities in neonates hatching from surfaced sterilized eggs (no direct contact with soil) were compared to gut communities in third instar larvae originating from the same population of adults. For this, ASVs from midgut and hindgut compartments in third instar larvae were merged followed by ASV filtering as described above.

Diversity metrics were estimated using the q2-diversity plugin with a resampling depth of 45,591 and 11,781 sequences per sample for the analysis of neonates and compartments (i.e., midgut and hindgut of third instar JB, and associated soil), respectively. To evaluate within-sample diversity (α-diversity), observed ASVs (richness), evenness (Pielou, 1966), Shannon diversity (richness and evenness) (Shannon and Weaver, 1949), and Faith's phylogenetic distance (phylogenetic diversity) (Faith, 1992) were calculated for each sample. To evaluate relationships among samples (β-diversity), four traditional methods and an approach that considers the compositional nature of the data were employed. The Jaccard distance metric (Jaccard, 1901) reflects the absence/presence of ASVs (unweighted), whereas Bray-Curtis dissimilarity (Bray and Curtis, 1957) considers the abundance of ASVs (weighted). Unweighted/weighted UniFrac distance (Lozupone and Knight, 2005; Lozupone et al., 2007) also considers phylogenetic relatedness. Additionally, β-diversity between compartments was calculated with DEICODE (matrix completion based and robust Aitchison principal component analysis) (Martino et al., 2019) *via* the q2-deicode rpca plugin. DEICODE was used because it considers the compositional nature of the data (Gloor et al., 2017), its capacity to handle datasets that include zeros, there is no need for rarefaction, and preservation of feature loadings which are linked to sample ordinations for further analysis. By using this combination of approaches to evaluate β-diversity, we were able to gain necessary insights, increase confidence in our biological interpretations, and prevent spurious interpretations.

Further statistical analyses were designed to characterize the influence of location and compartment on microbial diversity and to identify soil parameters that could potentially explain interactions between location and compartment.

Statistical analysis of α-diversity of the JB gut and soil microbiota was performed using R (version 3.6.1). Models were chosen based on how well the residuals met the assumptions of the models, with parametric analyses being preferred but only used when appropriate. Normality and homogeneity of variance of the residuals were tested using the Shapiro-Wilk test (stats-package) and Levene's test (car-package), respectively. To examine the influence of location, compartment (soil, midgut, and hindgut), and the interaction between these factors (location × compartment) on α-diversity, data were subjected to the aligned rank transform (ART) procedure, a non-parametric technique for conducting factorial ANOVA (ARTool package) (Wobbrock et al., 2011; Kay and Wobbrock, 2019). The interaction between location and compartment was decomposed using three separate procedures. First, differences between compartments were compared across all locations (across locations) using the contrast function (emmeans-package) to generate differences of differences, reporting Tukey-corrected for multiple comparisons *p*-values. The resulting pairwise comparisons were then grouped using the cldList function in rcompanion (version 2.3.25). This procedure allowed us to examine how changes in prokaryotic α-diversity between compartments varied from location to location by comparing the slopes between a given pair of compartments. Second, differences between compartments within a given location (within the location) were emphasized using non-parametric one-way ANOVA on ranks (i.e., Kruskal-Wallis *H*-test) with compartment (soil vs. midgut, soil vs. hindgut, or midgut vs. hindgut) as the independent variable and α-diversity as the dependent variable. This procedure allowed us to examine how α-diversity varied between compartments at each location independently. Lastly, variation in α-diversity within a given compartment (within compartment) was compared across locations using one-way ANOVA followed by Tukey's HSD, where the location was the independent variable and α-diversity was the dependent variable. This procedure allowed us to examine how α-diversity within a particular compartment varied at each location independently.

The influence of location, compartment, and their interaction (location × compartment) on variation in β-diversity was examined using a two-way permutational ANOVA (PERMANOVA, Adonis) (Anderson, 2001; Oksanen et al., 2018) with 999 permutations, in the q2-diversity plugin. For each PERMANOVA comparison, overall *F* statistics, *R*^2^, and *p*-values for the models were reported. Permutational analysis of multivariate dispersion (PERMDISP) (Anderson, 2001) was performed using the q2-permdisp plugin, with location or compartment as the main effect. PERMANOVA and PERMDISP were also used to examine each compartment independently to assess the influence of location on variation and dispersion of β-diversity, respectively. For each PERMADISP comparison, overall *F* statistics and *p*-values were reported.

The software QIIME 2 plugin wraps Emperor (Vázquez-Baeza et al., 2013) was used to visualize principal coordinate analyses (PCoA) generated using traditional β-diversity metrics and PCoA biplots generated using DEICODE. PCoA biplots comprised feature loadings (i.e., ASV contributions to variation along a given axis in the PCoA biplot) to reveal which taxa were driving the clustering observed in the ordination. The q2-qurro loading-plot plugin (Fedarko et al., 2020) was used to visualize feature loadings for axis 1 produced by DEICODE. In order to examine variation between key prokaryotic taxa across compartments, their natural log-ratios were compared using a Brown-Forsythe test (R version 3.6.1), with compartment as the independent variable and natural log-ratio of key taxa as the dependent variable. This analysis was followed by pairwise comparisons using a Bonferroni correction for multiple comparisons. Key taxa (sample and location prevalence ≥33% in all compartments) were selected from the three most prevalent orders in the prokaryotic core microbiota of each compartment and the orders driving most of the variation among compartments in the ordination space.

Core microbiota analyses were performed using bioinformatics tools implemented in MicrobiomeAnalyst (Dhariwal et al., 2017; Chong et al., 2020). Taxa with prevalence ≥0.6 (at the ASV level for neonates) or >0.19 (at the order level for compartments), at a minimum detection threshold of 1% relative abundance, were considered part of the core microbiota. These inclusive prevalence thresholds provided the opportunity to clearly visualize the sharing of key taxa across compartments at different detection thresholds—an approach we chose intentionally to facilitate comparisons with future studies. Heatmaps characterizing both the prokaryotic community in each sample at each location and core microbiota per sample type were visualized using ComplexHeatmap (version 2.2) (Gu et al., 2016) and circlize (version.4.8) (Gu et al., 2014). Heatmaps for the prokaryotic community included hierarchical clustering of features using Euclidean distance and the average method for orders and represented taxa with relative abundance ≥0.016% (118 sequences, ~1% of rarefaction at 11,781) among all samples. Boxplots were generated using the ggplot2 R package (Wickham et al., 2016).

Correlations between JB gut α-diversity and host soil physicochemical characteristics were analyzed using Spearman's (non-parametric) rank-order correlation test available in the PAST version 4.03 software (Hammer et al., 2001). Canonical correspondence analysis (CCA) was used to examine the relationships between ASV composition in each JB gut compartment (i.e., midgut, hindgut) and the physicochemical characteristics of their host soil environment. CCAs were performed using the cca() function of the R (version 3.6.1) package vegan (v2.5-7), with ASV tables and host soil physicochemical characteristics. An ANOVA-like permutation test (999) was used to assess the significance of constraints in the CCAs (anova.cca).

## Results

### Environmental Sourcing of JB Prokaryotic Gut Microbiota

**Figure 1:**
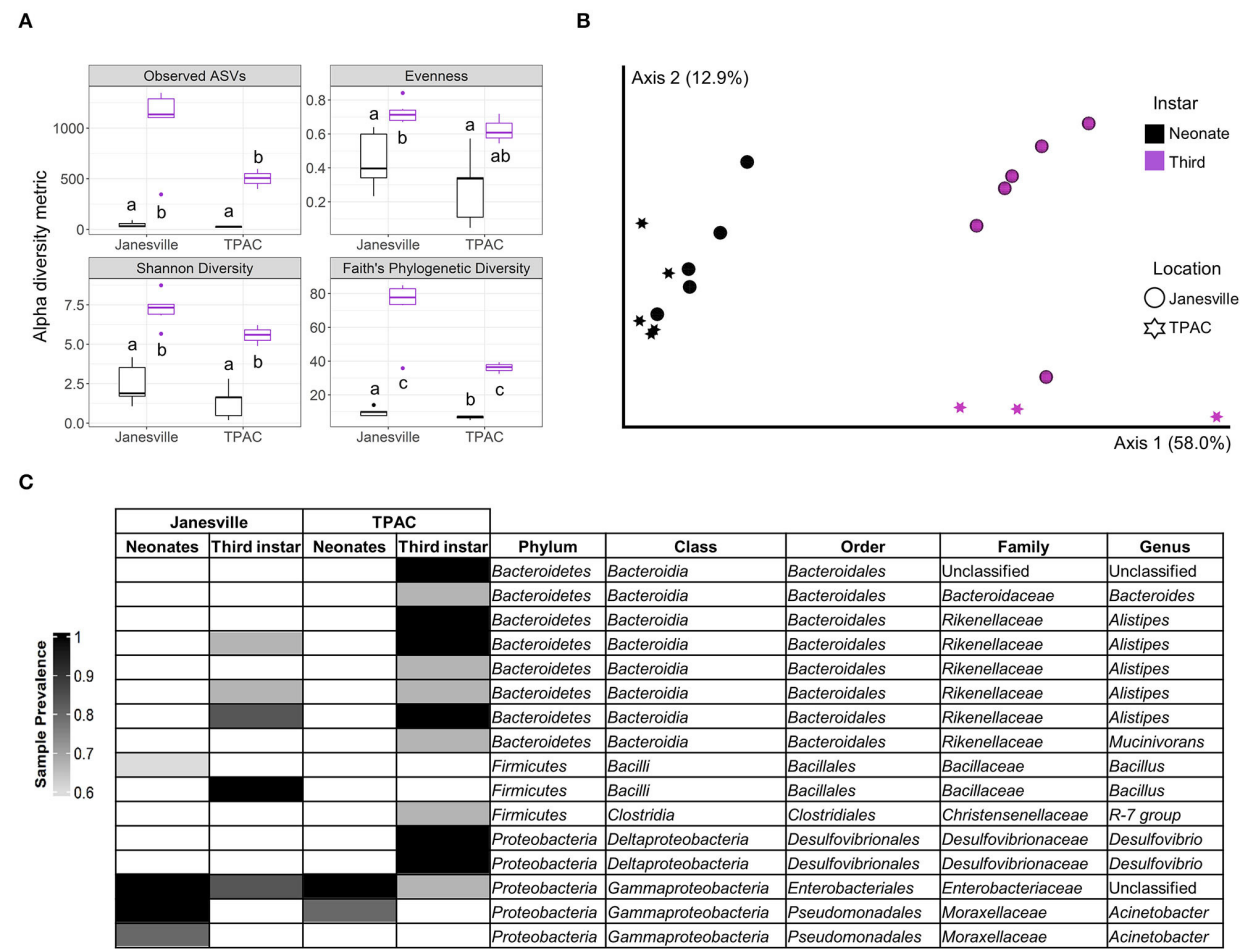
Comparison of gut prokaryotic microbiota of neonates eclosing from surface-sterilized eggs and field-collected third instar Japanese beetle (*Popillia japonica* Newman) larvae. Whole-gut microbial communities in neonates eclosed from surfaced sterilized eggs (black) were compared to the combined midgut and hindgut microbial communities in third instar larvae (purple) collected from the same locations. Variation in **(A)** α-diversity metrics (different letters mean statistically significant at α = 0.05, Kruskal–Wallis), **(B)** β-diversity, principal coordinate analysis for weighted UniFrac (PERMANOVA, pseudo-F_4, 19_ = 9.7, *p* = 0.001), and **(C)** core prokaryotic microbiota [amplicon sequence variants (ASVs) ≥0.01 relative abundance and sample prevalence ≥ 0.6] represented by a heatmap. All ASVs belonged to *Bacteria*.

Ten composite JB neonate gut samples (2 locations, *n* = 5) yielded 1,063,481 16S rRNA gene reads (82.4% of raw input, Supplementary Table 1) and 2,444 ASVs detected with.5 and 99.5% of ASVs belonging to *Archaea* and *Bacteria*, respectively. Neonate gut communities were significantly less rich, less diverse, and less even (Kruskal-Wallis, *p* < 0.05) than third instar larval communities (Figure 1A). Neonate gut communities from the two populations were similar (PERMANOVA, Weighted UniFrac/Bray Curtis, *p* = 0.125), but both were significantly different from gut communities of third instar larvae collected from the same population (PERMANOVA, Weighted UniFrac/Bray Curtis, *p* < 0.026; Figure 1B). The core gut microbiota of neonates from both locations consisted mainly of *Enterobacteriales* (*Enterobacteriaceae*) and *Pseudomonadales* (*Moraxellaceae*) (Figure 1C). Although ASVs from *Enterobacteriales* persisted in third instar larvae, ASVs from *Pseudomonadales* did not persist at ≥0.01 relative abundance. The core gut microbiota of neonates from Janesville contained one additional ASV classified as *Bacillales* (*Bacillaceae*), which did not persist at ≥0.01 relative abundance in third instar larvae from the same location.

### Gut Microbiota in Third Instar JB Larvae and Associated Soil

Sixty-three samples (7 locations ×3 compartments × and 3 biological replicates) yielded 4,209,746 reads (78.1% of raw input, Supplementary Table 1) for downstream analysis. Overall, 8,418 prokaryotic ASVs were detected with.4 and 99.6% belonging to *Archaea* and *Bacteria*, respectively (Supplementary Table 2). Samples were rarefied at the lowest library size (11,781), resulting in >81% coverage across all compartments compared to the unrarefied dataset (Supplementary Table 2). Rarefaction plots indicated sufficient sequence coverage (Supplementary Figure 2).

Between-compartment distribution of prokaryotic ASVs varied between locations (Supplementary Figure 3) with a few patterns being discernable. Most of the prokaryotic ASVs present in the soil (79.3 ± 12.1%) and hindgut (88 ± 8.7%) were unique to those compartments, while only 36.1 ± 9.8% of prokaryotic ASVs were unique to the midgut. The soil hosted a considerable fraction of prokaryotic ASVs (19.7 ± 11.8%) that were also found in the midgut, and these ASVs constituted a substantial portion of the midgut community (46.1 ± 17.6%). In contrast, only 2.3 ± 1.4% of prokaryotic ASVs found in the soil (with a relative abundance of 7 ± 4.5% in the soil) were also present in the hindgut with these ASVs constituting 5.7 ± 3.7% of hindgut ASVs (with a relative abundance of 12.1 ± 8.9% in the hindgut).

#### Core Microbiota

**Figure 2:**
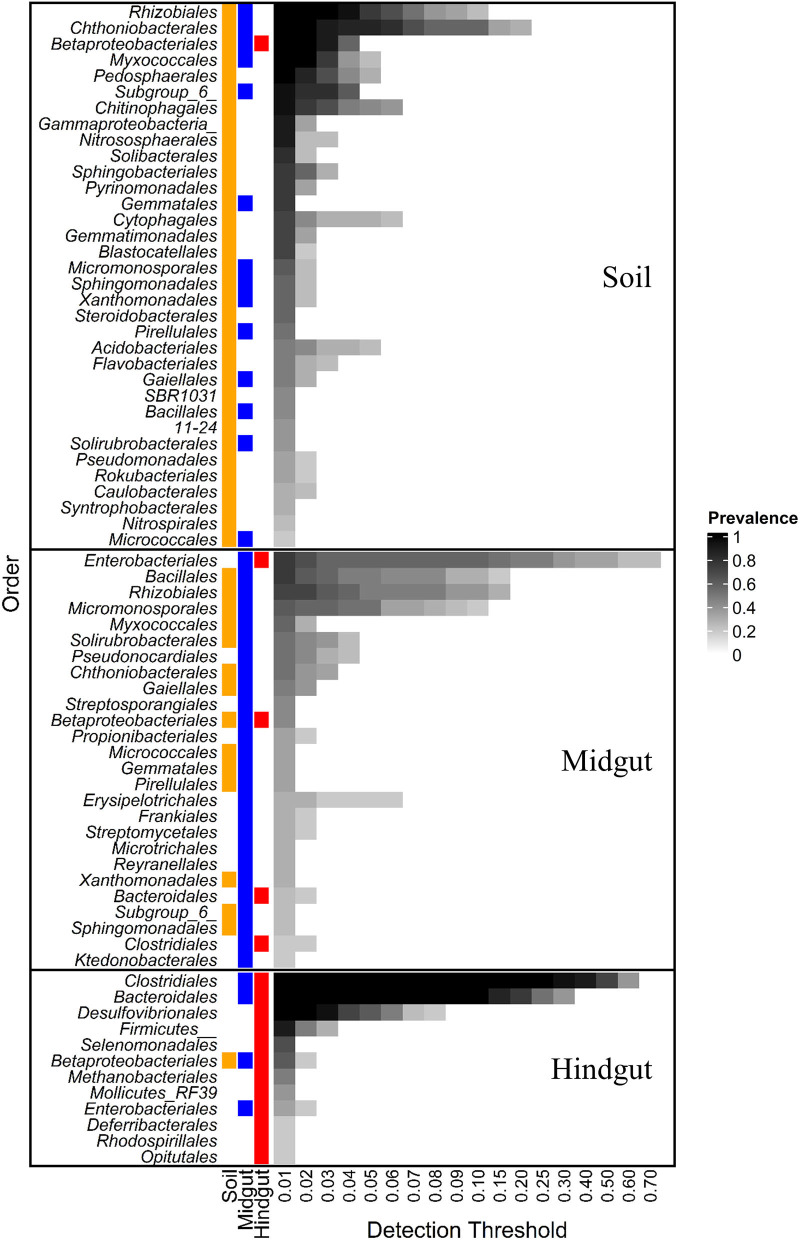
Core prokaryotic microbiota at different detection thresholds of relative abundance across compartments: midgut and hindgut from third instar larvae of the Japanese beetle (*P. japonica*) and associated soil. Taxa at the order level with sample prevalence >0.19 (>4 out of 21 samples) are presented. Colors show the presence of each taxon in different compartments at ≥0.01 relative abundance. Notice that *Betaproteobacteriales* are present in the three compartments. Taxa are presented at Order rank when available, otherwise, Class or Phylum rank are presented as indicated by Class/Phylum name followed by one or two underscore symbols, respectively. For taxonomic affiliation at Order, Class, or Phylum rank refer to Supplementary Table 3.

At order rank, the influence of geographic location on the composition of the prokaryotic gut community was evident when either the entire community (Supplementary Figure 4) or the core microbiota (Figure 2) were considered. The core microbiota in soil, midgut, and hindgut was composed of 34, 26, and 12 prokaryotic orders, respectively. *Betaproteobacteriales* were the only order present in the core microbiota of all three compartments, with relative abundance decreasing from soil to gut, and family level representation shifting in transit through the gut. Within *Betaproteobacteriales, Nitrosomonadaceae* and *Burkholderiaceae* represented the most abundant families in both soil (36.5 ± 7.3 and 27 ± 9.2%, respectively), and midgut (15.2 ± 11.2 and 47.4 ± 17%, respectively), whereas the families *Rhodocyclaceae, Burkholderiaceae*, and an unclassified ASV represented the most abundant families in the hindgut (55.2 ± 26, 31 ± 20.3, and 12.8 ± 6.3, respectively).

More than half (53.8%, 14/26) of taxa in the midgut core microbiota were also present in the soil core, with *Bacillales* and *Rhizobiales* being the most prevalent (76 and 71%, respectively). The relative abundance of *Bacillales* increased 10X from soil to midgut with most (94.2 ± 9.8%) ASVs in the midgut belonging to the genus *Bacillus*. The relative abundance of *Rhizobiales* did not vary between soil and midgut, with the families *Xanthobacteraceae* (~48%) and *Rhizobiaceae* (~15%) being most prevalent.

Lastly, 11% of the orders (3/26) composing the JB midgut core carried over to the JB hindgut core, representing 40% of orders (3/12) composing the JB hindgut core. Aside from *Betaproteobacteriales, Enterobacteriales* (76 and 33% prevalence in midgut and hindgut, respectively), and *Clostridiales* (19 and 100% prevalence in midgut and hindgut, respectively), represented the main taxa carrying over from the midgut core to the hindgut core. The relative abundance of *Enterobacteriales* decreased from midgut (31.2 ± 27.8%) to hindgut (1.5 ± 2%), with all ASVs within the order belonging to the family *Enterobacteriaceae*. The relative abundance of *Clostridiales* increased from the midgut (3.6 ± 5.4%) to the hindgut (54.6 ± 8.7%), with *Ruminococcaceae* (35.5 ± 4.4%), *Christensenellaceae* (19.9 ± 3%), and *Lachnospiraceae* (15.8 ± 4.1%) being most prevalent in the hindgut.

#### 16S rRNA Gene α-Diversity

**Figure 3:**
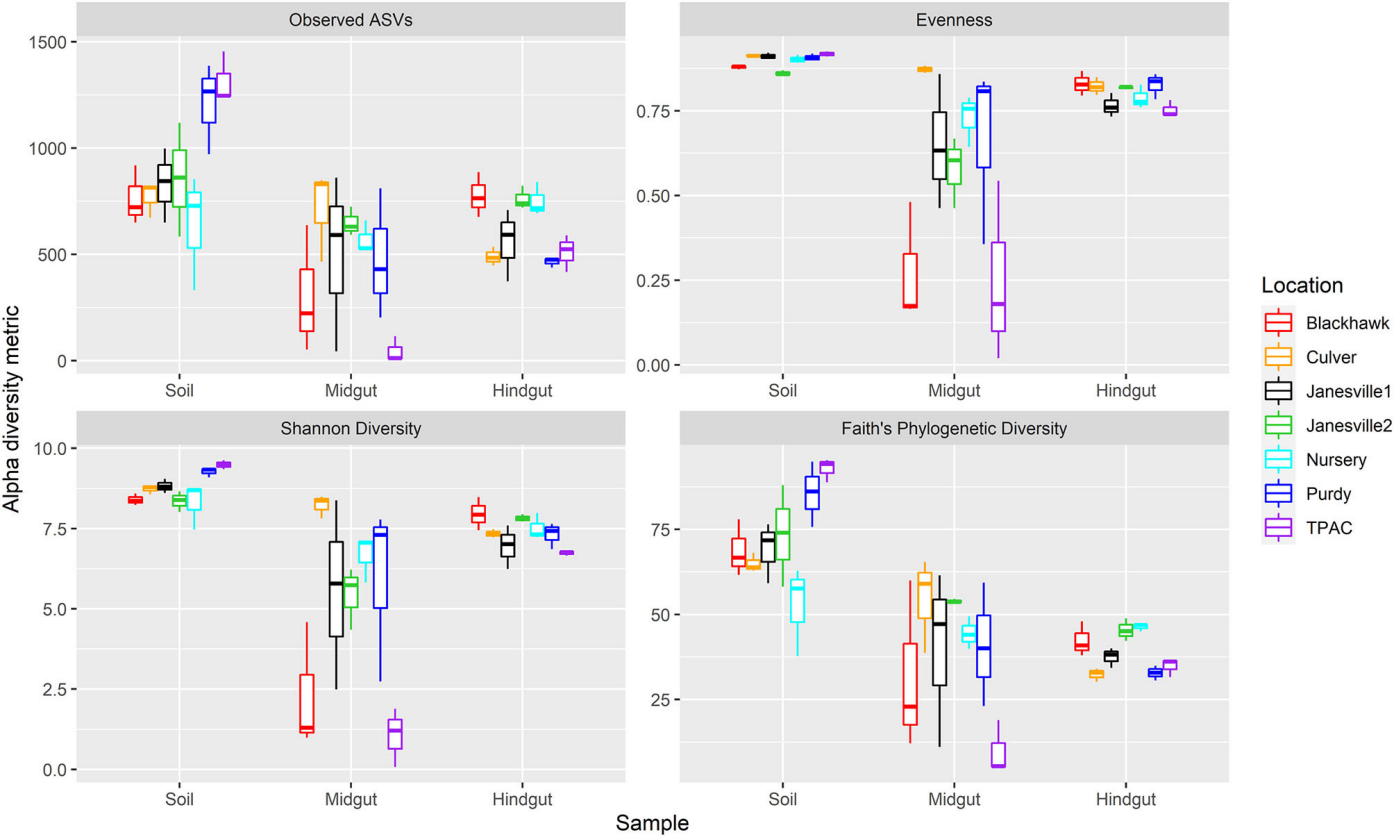
Alpha diversity for prokaryotic communities in guts from third instar Japanese beetle (*P. japonica*) larvae and associated soil. Boxplots show median, interquartile range, and 1.5 × the interquartile range per location. Regardless of the α-diversity metric, the influence of location on α-diversity varied with compartment (ART, location × compartment, *p* < 0.001). *n* = 3 for each compartment at each location. Refer to Supplementary Table 4 for detailed statistical significance.

Prokaryotic α-diversity within the three compartments varied with geographic location (Figure 3, ART, compartment × location interaction, *p* < 0.001, Supplementary Table 4). The interaction between compartment and location was characterized by differential changes to α-diversity in transit through the insect alimentary canal resulting in 1) variation between compartments at a given location (Supplementary Table 5) and 2) variation within a given compartment at different locations (Supplementary Table 6).

Although prokaryotic α-diversity was generally higher in the soil compared to the midgut, the magnitude of this difference varied across locations with the greatest differential change in observed ASVs and phylogenetic diversity in the agricultural system (TPAC). Although evenness and Shannon diversity were always greater in the soil compared to the midgut, observed ASVs and phylogenetic diversity were statistically similar at several locations. Samples collected at Purdy registered higher α-diversity in the soil, regardless of the α-diversity metric being examined, but observed ASVs and phylogenetic diversity of the JB larval midgut more closely resembled that of the Nursery soil after 7 d of exposure.

Similar patterns of change in prokaryotic α-diversity were observed between soil and hindgut compartments. Although evenness was always greater in the soil compared to the hindgut, these compartments shared similar observed ASVs, Shannon diversity, and phylogenetic diversity at one or more locations. Samples from Purdy revealed consistently higher α-diversity in the soil but observed ASVs, Shannon diversity, and phylogenetic diversity of the JB larval hindgut more closely resembled that of the Nursery soil after 7 d of exposure.

Changes in α-diversity between the midgut and hindgut compartments yielded more variable results with no single α-diversity metric showing a consistent trend. In fact, all three possible patterns of change in α-diversity were observed between these compartments (midgut > hindgut, midgut < hindgut, ND). Although no significant difference in α-diversity was apparent between midgut and hindgut communities at Purdy, observed ASVs and Shannon diversity of the JB larval hindgut were significantly higher compared to the midgut after 7 days of exposure to the Nursery soil.

#### 16S rRNA Gene β-Diversity

**Figure 4:**
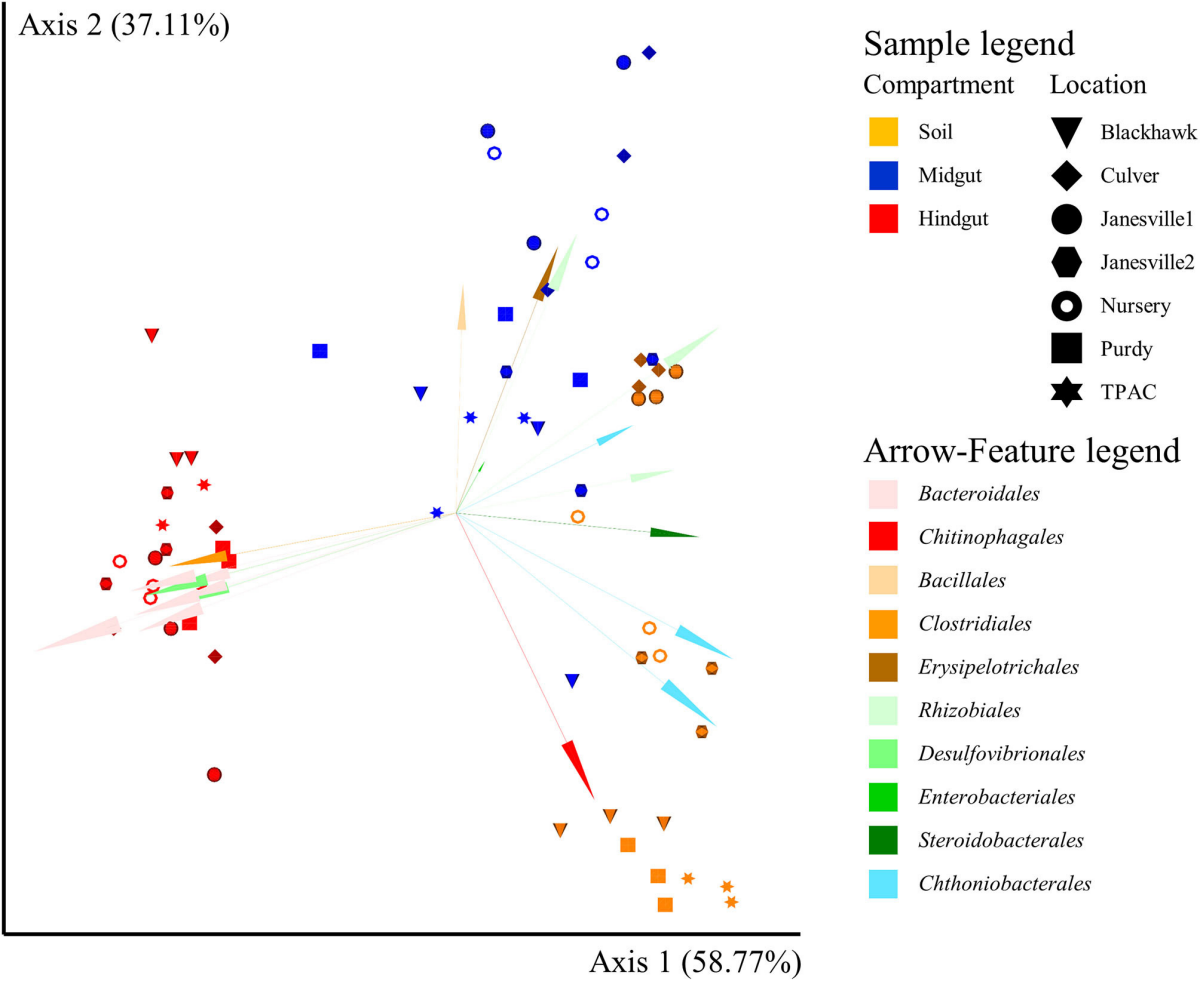
Compositional principal coordinate analysis (PCoA) biplot portraying beta diversity of prokaryotic communities in midgut and hindgut of third instar Japanese beetle (*P. japonica*) larvae and associated soil. The PCoA biplot was generated using DEICODE (Robust Aitchison PCA) (Martino et al., 2019) and visualized in Emperor (Vázquez-Baeza et al., 2013). Data points represent individual samples where symbol shape denotes location while symbol color denotes compartment (i.e., midgut, hindgut, or soil). The top 20 taxa driving differences in ordination space at the order rank are illustrated by the arrows. All taxa belong to *Bacteria*. The phyla within *Bacteria* are represented by specific hues, where *Bacteroidetes* (7) = red/pink, *Firmicutes* (3) = orange/brown, *Proteobacteria* (7) = green, and *Verrucomicrobia* (3) = blue. SILVA v132 database (Quast et al., 2012; Glöckner et al., 2017) was used for taxonomic classification.

Similar to α-diversity, the influence of location on β-diversity of the prokaryotic community (Figure 4, Supplementary Figure 5) varied with compartment, independent of the β-diversity metric being considered (the location × compartment interaction, *F* ≥ 2.4; df = 12, 42; *p* ≤ 0.001, *R*^2^ ≥ 0.138; Supplementary Table 7). Importantly, however, the compartment described a greater proportion of total variation in β-diversity, relative to location, by all five measures we considered. Microbial compositional profiles (Figure 4, Supplementary Figure 5) also revealed significant variation in dispersion among compartments independent of the β-diversity metric employed (*F* ≥ 8.3; df = 2, 62; *p* = 0.001, Supplementary Table 8). Hindgut communities were significantly less dispersed than midgut or soil communities (*F* ≥ 1.4; df = 1, 40; *p* ≤ 0.003, Supplementary Table 8), with soil communities being significantly less dispersed compared to midgut communities (*F* ≥ 11.4; df = 1, 40; *p* ≤ 0.007, Supplementary Table 8) when rarefied, qualitative (Jaccard, unweighted UniFrac), or quantitative (weighted UniFrac) β-diversity metrics were considered. No significant variation in dispersion of prokaryotic communities among locations was observed (*F* ≤ 0.6; df = 6, 62; *p* ≥ 0.366, Supplementary Table 8). Greater than 95% of the total variation in prokaryotic communities observed among compartments and locations was described by the first two axes of the PCoA compositional biplot generated using DEICODE (Figure 4). Prokaryotic communities in the hindgut clustered separately from the other two compartments along the first axis, with *Bacteriodales, Clostridiales*, and *Desulfovibrionales* primarily differentiating hindgut communities from communities in the other two compartments. Midgut and soil prokaryotic communities separated primarily along the second axis, and although these two communities displayed greater interspersion (shared features or ASVs), members of six prokaryotic orders appeared to differentiate them from each other; *Bacillales, Enterobacteriales, Erysipelotrichales*, and *Rhizobiales* (midgut), and *Chitinophagales*, and *Chthoniobacteriales* (soil). The PCoA biplot also revealed that variation between locations was largely captured along the second axis.

Among the key taxa (Supplementary Table 9), *Clostridiales* had the highest representation (2,106 ASVs) and was present at all locations and in most samples (≥95%). *Enterobacteriales* had the lowest representation (10 ASVs) but were likewise present at all locations and in most samples (≥95%). Similarly, *Bacillales* was represented by only 61 ASVs but members of this order were present in 100% of the samples. In contrast, *Desulfovibrionales* (22 ASVs) and *Bacteroidales* (47 ASVs) were detected in ≤ 47% of soil samples (≤ 71% of locations) but increased in transit through the alimentary canal (midgut ≥ 71% of samples, 100% of locations; hindgut 100% of samples). *Rhizobiales* (234 ASVs), *Betaproteobacteriales* (222 ASVs), and *Chthoniobacterales* (142 ASVs) were present in 100% of soil and hindgut samples, but only in 76, 90, and 95% of midgut samples, respectively.

**Figure 5:**
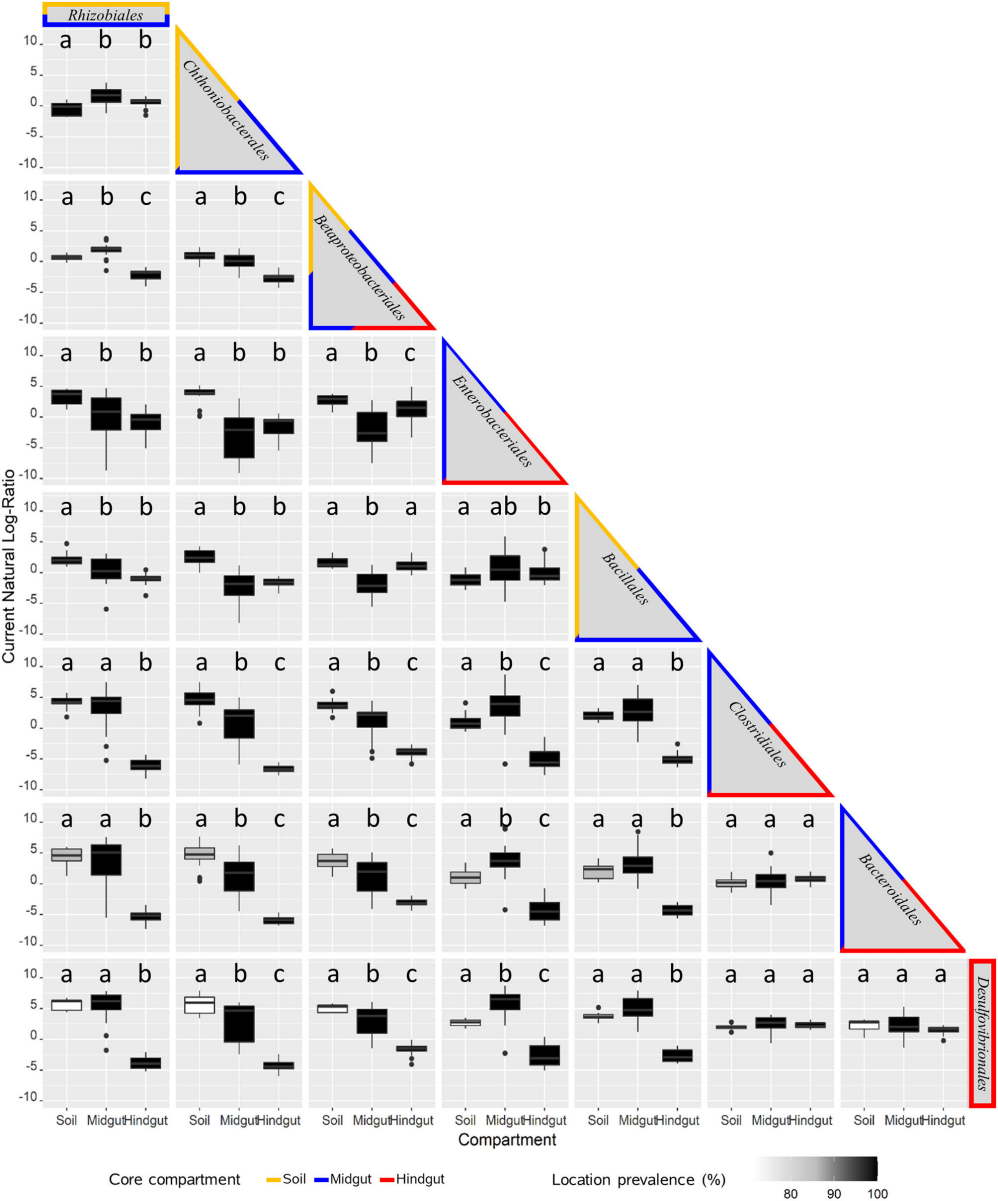
Pairwise analysis of key prokaryotic taxa variation across compartments: midgut and hindgut from third instar larvae of the Japanese beetle (*P. japonica*), and associated soil. The natural log-ratio of taxa for each sample was calculated using qurro (Fedarko et al., 2020), with taxa in columns as numerators, and the ones in rows as denominators. Taxa are presented in order of criteria for consideration (Supplementary Table 6), including the most prevalent and/or top taxa driving most of the difference among compartments. The border color identifies the presence of taxa in the core microbiota of the compartment for soil, midgut, and hindgut. The presence of taxa in locations (location prevalence %, *n* = 7) is represented by the color of the boxplot. Compartments with different lowercase letters indicate a significant difference at α = 0.05, Brown-Forsythe Test with Bonferroni correction.

Pairwise analysis of log-ratios between key taxa provided another way to describe variation across compartments and locations. This analysis resulted in 28 comparisons (Figure 5, Supplementary Table 10) with three broad patterns emerging. For 12 inter-taxa comparisons (42.9%), log-ratios varied significantly between all three compartments, with discernable variation in prevalence among locations observed in 50% of cases. For the second broad pattern, 12 inter-taxa comparisons (42.9%) revealed log-ratios that differed significantly in only one compartment compared to the other two, with a variation in prevalence among locations being discernable in 30% of these cases. The third pattern included 4 inter-taxa comparisons (14.3%), where no differences in the log ratios were observed across compartments.

To further investigate the interaction between location and compartment, variation in β-diversity of the prokaryotic communities in each compartment was explored independently across locations (Supplementary Table 11). These comparisons included soil management history (TPAC vs. naturally infested locations) and the transfer of larvae from one soil to another (Purdy vs. Nursery). When all locations were considered, the location was a significant predictor of β-diversity in all compartments (*F* ≥ 1.6, *p* ≤ 0.008), but it was only a significant predictor of dispersion in the hindgut (Bray-Curtis, *F* = 2.7, *p* = 0.007). Soil management history was a significant predictor of soil (Jaccard, Bray-Curtis, Unweighted UniFrac, and DEICODE, *F* ≥ 2.96, *p* ≤ 0.008), midgut (Jaccard and unweighted UniFrac, *F* ≥ 1.81, *p* ≤ 0.012), and hindgut (Jaccard, Bray-Curtis, Unweighted/weighted UniFrac, *F* ≥ 1.55, *p* ≤ 0.043) β-diversity, and soil (*F* ≥ 25.9, *p* ≤ 0.009) and hindgut dispersion. Prokaryotic β-diversity in soils from Purdy and Nursery was not significantly different (*F* ≤ 21.87, *p* ≥ 0.099), and transferring larvae from one soil (Purdy) to another (Nursery) did not appear to be a significant predictor of β-diversity in the JB gut communities (F ≤ 2.76, *p* ≥ 0.101). Further, location (Purdy vs. Nursery) of manipulated larval treatment was a significant predictor of dispersion in soil (Purdy < Nursery, weighted UniFrac, *F* = 0.27, *p* = 0.046), midgut (Purdy > Nursery, Bray-Curtis, *F* = 26.37, *p* = 0.043), and hindgut (Purdy > Nursery, Unweighted UniFrac, *F* = 0.84, *p* = 0.045).

### Influence of Host Soil on JB Gut Prokaryotic Communities

The physicochemical characteristics of the soils sampled in this study (Table 1) revealed a relatively high degree of heterogeneity typical of managed soils (Jasinska et al., 2006) and accounted for a significant portion of the variation in α-diversity and community composition within the alimentary canal of the third instar JB larva. In the midgut, evenness and Shannon diversity were positively correlated with soil texture (% sand) (Table 2). In the hindgut, evenness was negatively correlated with cation exchange capacity (CEC), and pH, whereas Shannon diversity was negatively correlated with water holding capacity (WHC).

**Table 2:** Spearman's (non-parametric) rank-order correlations between α-diversity metrics in gut prokaryotic microbiota in Japanese beetle (*P. japonica* Newman) larvae and host soil physicochemical characteristics across locations.

	**Midgut**								**Hindgut**							
	**Observed ASVs^a^**		**Evenness**		**Shannon diversity**		**Faith's PD^b^**		**Observed ASVs^a^**		**Evenness**		**Shannon diversity**		**Faith's PD^b^**	
**Soil variable**	***r_s_***	***p***	***r_s_***	***p***	***r_s_***	***p***	***r_s_***	***p***	***r_s_***	***p***	***r_s_***	***p***	***r_s_***	***p***	***r_s_***	***p***
CEC^c^	−0.236	0.303	−0.299	0.188	−0.350	0.120	−0.260	0.256	−0.083	0.722	**−0.448**	**0.042**	−0.342	0.129	−0.075	0.748
OM^d^	0.042	0.858	0.157	0.498	0.093	0.688	0.004	0.986	−0.298	0.190	−0.212	0.356	−0.321	0.155	−0.413	0.063
pH	−0.236	0.303	−0.271	0.234	−0.338	0.134	−0.232	0.312	−0.232	0.312	**−0.436**	**0.048**	−0.405	0.069	−0.201	0.383
Sand	0.315	0.164	**0.559**	**0.008**	**0.502**	**0.020**	0.226	0.324	−0.020	0.932	−0.052	0.824	−0.060	0.798	−0.054	0.818
WHC^e^	−0.362	0.107	−0.362	0.107	−0.389	0.081	−0.334	0.139	−0.389	0.081	−0.338	0.134	**−0.444**	**0.044**	−0.358	0.111

a *ASVs, Amplicon sequence variants;*

b *Faith's PD, Faith's phylogenetic distance;*

c *CEC, Cation Exchange Capacity (meq/100 g);*

d *OM, Organic matter percentage;*

e *WHC, Water Holding Capacity at 1/3 Bar. Boldface values indicate significant correlation (p ≤ 0.05)*.

**Figure 6:**
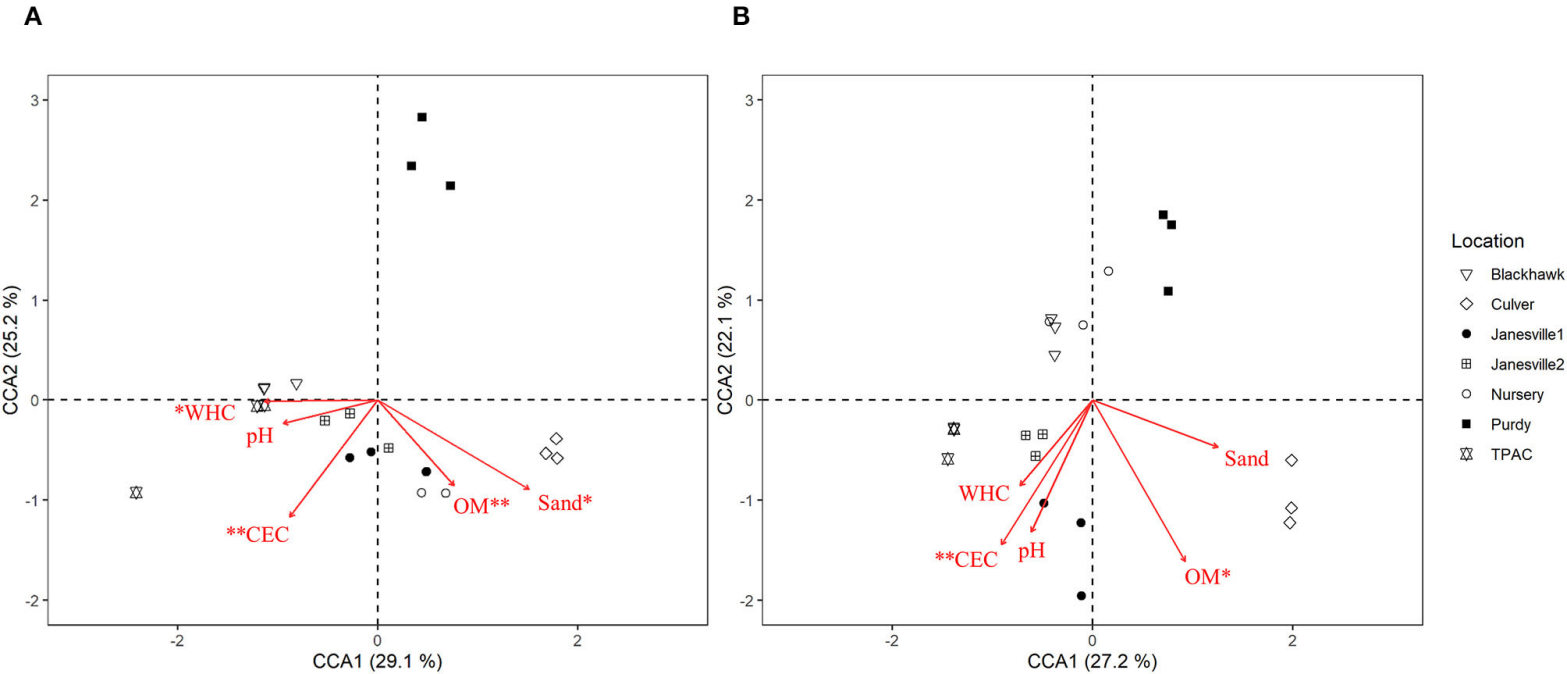
Canonical correspondence analysis (CCA) calculated based on a Chi-square dissimilarity matrix of prokaryotic communities at the ASV rank in the midgut **(A)** and hindgut **(B)** of third instar Japanese beetle (*P. japonica*) larvae and host soil physicochemical parameters. Vectors show soil variables: cation exchange capacity (CEC), % organic matter (OM), pH, % sand (Sand), and water holding capacity (WHC). Significant soil variables are presented with two (*p* ≤ 0.01) or one (*p* ≤ 0.05) asterisks.

Canonical correspondence analysis (CCA) was used to examine the extent to which host soil physicochemical characteristics corresponded with the 16S rRNA gene ASV composition of the JB midgut (Figure 6A, CCA-ANOVA: *p* < 0.001, *n* = 999), and hindgut (Figure 6B, *p* < 0.001, *n* = 999). These soil characteristics (constraints) explained 36% of the total variation in the prokaryotic composition of the midgut community across locations with the first two CCA axes explaining 54% of that variation. CEC, % organic matter, soil texture, and WHC were significant predictors of midgut prokaryotic composition. Soil characteristics (constraints) explained slightly less (29%) of the total variation in JB hindgut ASV composition across all locations with the first two CCA axes explaining 49% of that variation. CEC and % organic matter were significant predictors of hindgut prokaryotic composition.

## Discussion

The invasive Japanese beetle (*P. japonica* Newman) (JB) spends approximately 75% of its life as soil-dwelling larvae feeding on plant roots and other organic material that have low nutritive values (Smith, 1922). During the 9-month larval stage, copious amounts of soil pass through their digestive system (Swingle, 1931), exposing the larvae to a diversity of microorganisms (Fierer, 2017; Jansson and Hofmockel, 2018). Despite their sustained exposure to such diverse reservoirs of potential gut symbionts, the dynamics of JB gut-microbial recruitment remain poorly understood. No previous studies aiming to discriminate between transient and core microbes associated with the JB larval host have been reported. Efforts to clarify the relative importance of vertical vs. horizontal transmission in structuring the JB gut microbial consortium are also lacking in the literature. By comparing gut prokaryotic communities associated with JB larval hosts over a range of soil types and geographies, we differentiated between core resident and transient microbiota in the larval gut and clarified the potential of vertical transmission for structuring these communities.

Although there are no defining criteria for determining which members of the microbial community should be considered as core microbiota (Risely, 2020; Neu et al., 2021), we chose rather inclusive prevalence thresholds for this study (≥0.6 or >0.19 for neonates or third instar larvae and associated soils, respectively). This approach was implemented intentionally in order to facilitate the identification of patterns in microbial frequencies across compartments and provide a foundation for building a deeper understanding of how variable environmental conditions may influence the JB-gut microbiota system. In this sense, our thresholds provide a somewhat larger pool of potential candidates for investigating microbial ecology and function. However, because this approach incorporated both prevalence and detection thresholds, our results should also be more easily comparable to future studies in the field, even when different prevalence and detection thresholds are selected.

### Environmental Sourcing of JB Prokaryotic Gut Microbiota

To assess the degree to which environmental sourcing shapes the JB larval gut microbial community, we analyzed gut microbial communities of neonates taken from two, spatially distant populations. Typically, vertical transmission through the insect egg takes place transovarially, *via* egg smearing of the chorion during oviposition, or through egg capsule transfer (Kucuk, 2020; Nyholm, 2020), but such strategies have not been described in JB or related scarabs. Further, in JB and most other beetles, parental care of offspring as a vertical transmission strategy is limited to the selection of a suitable location for oviposition (Crowson, 1981c). Our approach of surface sterilizing eggs prior to the eclosion of neonates should only have eliminated the vertical transfer of microbes through egg smearing. It did not preclude the potential for vertical transfer of microbes within the egg capsule itself. Without the influence of external environmental factors, the presence of microbes in the neonate's gut likely indicates that at least a small measure of maternal inheritance does take place (Kucuk, 2020), although this still remains to be more carefully quantified. We detected a somewhat depauperate prokaryotic community in the alimentary canal of neonates, with those communities being much less rich and diverse than those of third instar larvae exposed to the highly diverse soil environment. Similar trends have been observed in termites (Diouf et al., 2015). However, our results stand in contrast to previous studies. Chouaia et al. (2019) reported JB gut microbial communities of similar richness and diversity across all three larval instars, but each instar was collected from the field where they were in direct contact with the soil. In dung beetles (Coleoptera: Scarabaeidae) (Shukla et al., 2016; Suárez-Moo et al., 2020) and lepidopteran insects (Chen et al., 2016), whole egg microbial communities were more diverse than those present in the gut of later instar larvae. In light of our findings, it appears likely that, although a modest gut microbial community may be maternally transmitted to JB larvae inside the egg, that community is rapidly transformed once larvae begin feeding in soil or near plants.

To further support the idea that JB larvae receive only a minimal and short-lived transfer of maternal gut microbiota, three of the four ASVs detected in neonate's guts were not detected in the guts of third instar larvae. For instance, *Acinetobacter* sp. was present in neonates, but not in the third instar from Indiana or Wisconsin. This genus is comprised of strictly aerobic gram-negative bacteria commonly found in environmental samples (Jung and Park, 2015; Van Dexter and Boopathy, 2019 #406) and has been implicated as a vertically transmitted symbiont in dung beetles (Coleoptera: Scarabaeidae) (Hammer et al., 2016). In fact, *Acinetobacter* sp. was the most abundant operational taxonomic unit (OTU) in egg and whole-body samples of the adult dung beetle *Euoniticellus intermedius* (Shukla et al., 2016). Another report describes the presence of *Acinetobacter* sp. on the surface of *Stomoxys calcitrans* (Diptera: Muscidae) eggs, where it was required to ensure larval survival and development (Lysyk et al., 1999). Functions related to *Acinetobacter* sp. in insects have been associated with detoxification (Van Dexter and Boopathy, 2019), production of antiparasitic compounds (Lysyk et al., 1999), and insecticide metabolism (Malhotra et al., 2012). *Acinetobacter* has also been identified as a potential reservoir of antibiotic resistance genes (Malhotra et al., 2012; Tian et al., 2016). Given its potential physiological importance to the host insect, maternal transfer of *Acinetobacter* could provide ecological benefits to vulnerable neonate JBs that may be less critical as the larvae and associated microbiota develop.

### Gut Microbiota in Third Instar JB Larvae and the Associated Soil

To characterize how variation in soil environments influences the larval prokaryotic microbiota, we analyzed gut microbial communities in samples collected from seven locations across Indiana and Wisconsin, USA. Larval gut communities were distinct in both composition and relative abundance from that of their respective host soil communities. However, variation in soil microbial communities across the locations we sampled did influence larval communities, with the extent of this impact depending on the gut compartment (i.e., midgut or hindgut). Still, despite the variability between locations, a core microbiota for each compartment was identifiable indicating that the compartment has an outsized impact on the composition of the prokaryotic community and that the impact of location is a second-order concern. Results indicate a clearly defined core for both soil and hindgut with several microbial taxa being highly prevalent as long as thresholds for detection are relatively sensitive. In contrast, the midgut core appears to be less defined, constituting a broader assemblage of moderately prevalent microbial taxa, consistent with a region in transition between the soil and hindgut.

As with other scarabs, the unique physicochemical conditions and resources present in the guts of JB larvae (Chouaia et al., 2019) likely provide microenvironments suitable for microbial recruitment (Lemke et al., 2003; Huang et al., 2010). We observed a paring down of ASVs from the soil in transit through the alimentary canal, where about half (46.1 ± 17.6%) of ASVs in the midgut and only a small fraction (5.7 ± 3.7%) in the hindgut were detectable in the soil. Similarly, Chouaia et al. (2019) found that 37% of microbial species (defined as OTUs) present in whole gut samples from JB larvae were derived from the soil microbiota. Additionally, only a tiny fraction (≤ 0.7%) of ASVs present in the third instar larval guts were detected in the guts of neonates eclosed under sterile conditions. The source of the remaining larval gut ASVs remains to be elucidated. Aside from the soil, JB larvae could potentially recruit microbes from the non-soil components of the rhizosphere, specifically from the rhizoplane and endorhizosphere (Hacquard et al., 2015; Kawasaki et al., 2016), which were not interrogated in the present study. Additionally, microbes transmitted vertically during oviposition through egg smearing or other unknown mechanisms could not be ruled out by our experimental approach.

Although JB larvae are exposed to a diverse prokaryotic assemblage in different soil environments, we were able to assemble a core prokaryotic microbiota in the JB gut. This suggests that gut microbiota is not composed entirely of transient microbial associates and that symbiotic associations are important. In fact, transferring larvae from one soil to another did not alter microbial diversity in the larval gut over the short term, although changes in gut community dispersion potentially reflected a community in transition resulting from exposure to a novel soil community. In general, microbiota in host soils resemble those commonly found in the rhizosphere of turfgrass (Allan-Perkins et al., 2019; Azeem et al., 2020) and other grass-dominated ecosystems (Bergmann et al., 2011; Naylor et al., 2017), with soil microbial communities being richer and more diverse compared to the JB gut. Such decreases in microbial richness and diversity between soil and gut have been previously reported in JB (Chouaia et al., 2019) and other scarab larvae (Egert et al., 2003; Andert et al., 2010; Arias-Cordero et al., 2012), further supporting the notion that compartment drives variation in community composition to a greater extent than location.

Even among prokaryotic taxa that were shared by all three compartments (soil, midgut, and hindgut) at the order rank, the composition of lower rank taxa varied in transit through the gut. Lower rank representation of *Betaproteobacteriales* shifted from *Nitrosomonadaceae* and *Burkholderiaceae* being most abundant in soil and midgut to *Rhodocyclaceae* and *Burkholderiaceae* being more abundant in the hindgut. *Nitrosomonadaceae* comprise a monophyletic phylogenetic group whose cultivated representatives are aerobic lithoautotrophic ammonia oxidizers (Prosser et al., 2014) that have been identified in nearly all soils (Prosser et al., 2014). A higher prevalence of ammonia-oxidizing prokaryotes in parent soils compared to the gut of scarab larvae (Scarabaeidae: Coleoptera) has been previously reported (Majeed et al., 2014). *Burkholderiaceae* are phenotypically, metabolically, and ecologically flexible and versatile (Coenye, 2014) and symbiotic associations with eukaryotic organisms, including insects have previously been documented (Kaltenpoth and Flórez, 2020). Symbionts within the genus *Burkholderia* are environmentally sourced by the insect, and these symbionts may provide nutritional benefits, detoxifying capabilities, and protection against pathogenic fungi (Kaltenpoth and Flórez, 2020). Members of the *Rhodocyclaceae* have previously been found in many habitats, including in the hindgut of scarab larvae (Huang and Zhang, 2013), and other species that degrade a wide range of carbon sources through propionic acid fermentation (Oren, 2014). Considering the wide range of potential metabolic and ecological benefits provided by *Betaproteobacteriales*, it is not surprising that different conditions found in soil and gut compartments offered opportunities for differentiation among the families within this order.

The prevalence of core prokaryotic community members within the larval midgut varied with location, suggesting an extrinsically-mediated, transient community that varied with the surrounding soil environment, including different soil management history. This phenomenon has also been observed in other scarabs (Zhang and Jackson, 2008; Andert et al., 2010). In fact, in our study, 54% of microbial orders present in the midgut core were also present in the soil core, of which *Bacillales* (mainly *Bacillus* spp.) and *Rhizobiales* were most prevalent (76 and 71%, respectively). At the ASV level, however, three (19%) features present in the midgut core were also present in the soil core, corresponding to a *Rhizobiales* (*Xanthobacteraceae, Bradyrhizobium*), a *Chthoniobacterales* (*Chthoniobacteraceae*), and an *Enterobacteriales* (*Enterobacteriaceae*). In contrast, only one (2%) ASV present in the hindgut core was also present in the soil or midgut core (an *Enterobacteriales, Enterobacteriaceae*). *Bacillus* spp. are predominant in the guts of soil invertebrates, where they contribute to different stages of lignocellulose degradation under anoxic conditions (König, 2006). Furthermore, bacilli inhabiting the guts of scarab beetle larvae may be capable of fermentative carbohydrate metabolism and iron reduction within the gut and the soil environment (Hobbie et al., 2012). Rhizobia was the second most prevalent taxa in the JB midgut and previous studies indicate that *Rhizobiales* is among the most prevalent orders in the core microbiota shared between host soil and all JB developmental stages (Chouaia et al., 2019). Rhizobia also occur in the digestive systems of other herbivorous insects (Fröhlich et al., 2007; Russell et al., 2009; Pierce and Berry, 2011; Johnson and Rasmann, 2015), where they might regulate nitrogen balance in the gut. *Enterobacteria* (*Enterobacteriaceae*) was the most prevalent family in the midgut core. Bacteria in the family *Enterobacteriaceae* have been described in the alimentary canal of earthworms, where they feed on mucus- and plant-derived saccharides, producing succinate as a fermentation byproduct (Wüst et al., 2011). These findings indicate that the most prevalent microbes in the core microbiota of the JB midgut likely contribute nutritional benefits, aiding in the metabolism of carbohydrates (i.e., lignocellulose), and regulating nitrogen balance.

The consistent 100% presence of specific taxa within an insect species likely indicates a coevolved, mutually beneficial association (Ebert et al., 2021). As observed in other scarab larvae, the core prokaryotic community of the JB hindgut was more conserved compared to that of the midgut (Egert et al., 2005; Pittman et al., 2008; Zhang and Jackson, 2008; Andert et al., 2010). Members of *Clostridiales* and *Bacteroidales* are commonly found as part of the core microbiota in the digestive tract of scarab larvae (Andert et al., 2010; Huang and Zhang, 2013; Shelomi et al., 2019; Ebert et al., 2021) and other animals, including termites (Scharf and Peterson, 2021), ants (Lee et al., 2008), humans (Hacquard et al., 2015), and an assortment of mammalian hindgut fermenters (O'Donnell et al., 2017). Members of *Clostridiales* and *Bacteroidales* have a potential role in the degradation of plant roots and organic matter consumed by grass grub larvae (Zhang and Jackson, 2008) as these microbial orders include a wide range of anaerobic bacteria capable of fermenting sugars and more complex molecules, including cellulose, hemicellulose, pectin, and polysaccharides (Lynd et al., 2002; Hanreich et al., 2013; Foster et al., 2017). *Desulfovibrio* spp. comprises a group of obligate anaerobic bacteria that obtain their energy mainly by respiratory sulfate reduction (Garrity et al., 2005). *Desulfovibrio* spp. have been described in the digestive tract of scarab larvae (Arias-Cordero et al., 2012; Ziganshina et al., 2018; Shelomi et al., 2019; Ebert et al., 2021) and other insects, including termites (Kuhnigk et al., 1996) and cockroaches (Gontang et al., 2017). In scarab larvae, *Desulfovibrio* spp. may aid in sulfate metabolism, specifically by decreasing sulfate concentration between midgut and hindgut (Egert et al., 2005), and in the generation of acetate (Arias-Cordero et al., 2012). Overall, the prevalence of these microbes in the hindgut core microbiota may suggest important contributions to sulfate metabolism, as well as the fermentation of sugars and complex molecules present in the soil matrix.

Differences in key prokaryotic taxa between compartments were largely driven by dominant, high relative abundance taxa. These differences may reflect the capacity of taxa to exploit different niches within the alimentary canal (Rivett and Bell, 2018), where the insect may exploit their functional contributions. However, low relative abundance taxa that were not considered part of the core also varied between locations. These microbes may represent a substantial, potentially redundant pool of genetic resources that could be activated under suitable conditions (Jones and Lennon, 2010; Liang et al., 2020). The metabolic state (i.e., active, dormant) of both high and low relative abundance taxa, their metabolic contributions, and the nature of their interactions with other microbial constituents remains understudied. Obtaining this information could advance our ability to meaningfully interrogate insect gut microbiota, its plasticity, and microbial influences on insect health and invasive capacity.

We further searched for soil physiochemical correlates corresponding with α-diversity and community composition in third instar larval guts. Although underexplored in relation to JB gut microbial communities, soil characteristics such as texture and moisture may affect JB oviposition, egg, and larval survival (Allsopp et al., 1992). Additionally, JB larvae require soil organic matter for growth (Fleming, 1972). Whereas, soil characteristics have the potential to influence the composition of soil microbiota (Fierer and Jackson, 2006; Docherty et al., 2015; Delgado-Baquerizo et al., 2017; Fierer, 2017), our findings indicate that these characteristics are more tightly correlated to microbial communities in the midgut than in the hindgut. Notably, the prokaryotic composition of the JB gut did not vary with soil pH even though the pH of soils included in this study ranged from 5 to 7.3. Although a wider range of soil pH may be required to detect any impact on microbial gut constituents (Fierer, 2017), the digestive secretions associated with the JB gut possess a strong buffering capacity, whereby pH within the digestive tract remains relatively constant regardless of soil pH (Swingle, 1931). Given the important contribution of soil-derived microbes to the JB larval gut microbiota, soil environmental factors particularly, soil cation exchange capacity, organic matter, water holding capacity, and soil texture could influence larval gut microbiota directly by mediating the pool of available microbial symbionts. But, because these relationships were more pronounced in the midgut than in the hindgut, observed correlations may indicate only an indirect effect—a transient microbial footprint of the soil community that varies in importance and complexity as it passes through the gut. Our findings are not definitive in this regard.

In summary, this study elucidated that the diversity and composition of microbial communities in the midgut and hindgut of JB third instar larvae varied across locations in association with relatively large variation in host soil characteristics and soil management history. Our results indicated that, as previously reported in other scarab larvae, midgut microbiota contains more transient communities that vary with the surrounding soil environment while hindgut microbiota is more conserved. As such, defining a core microbiota within the midgut required a more inclusive prevalence threshold capable of circumscribing a broader assemblage of moderately prevalent microbial taxa. The source of a large proportion of JB gut microbes and additional potential mechanisms of vertical transmission remains to be elucidated. Further studies are clearly needed to understand the metabolic capabilities and ecology of microbial taxa inhabiting the gut of root-feeding scarab larvae. Because we employed a DNA-based method to address our objective of characterizing JB gut and soil microbial communities, the differentiation of viable and active taxa, their absolute abundances, specific locations within the digestive tract (e.g., *Acinetobacter*), and the role of other microbial communities relevant in insects (e.g., fungal, protists) remain unexplored. Such information can contribute to understanding the roles of soil and gut microbes in the physiology of invasive insects and services provided by microbial symbionts, as well as symbiont contributions to enhance the ability of the host to adapt to new environments. Such knowledge could also be used to identify new microbial targets for the management of highly invasive species such as JB. Finally, the influence of JB larval infestation on soil microbial communities and biogeochemical processes remains to be explored; this represents an important upcoming priority that will be enabled by the results presented here.

## Data Availability Statement

The original contributions presented in the study are publicly available. This data can be found at: https://www.ncbi.nlm.nih.gov/bioproject/, PRJNA798077.

## Author Contributions

DR designed the study. DR and HA-A performed the experiments, with the assistance of MS and RT. HA-A and DR wrote the manuscript with contributions from co-authors. All authors read the article and approved the submitted version.

## Funding

This study was supported by the Agricultural Science and Extension for Economic Development program (AgSEED) at Purdue University College of Agriculture, and the United States Department of Agriculture, National Institute of Food and Agriculture Research, Agriculture, and Food Research Initiative (Award No. 2018-67013-28062).

## Conflict of Interest

The authors declare that the research was conducted in the absence of any commercial or financial relationships that could be construed as a potential conflict of interest.

## Publisher's Note

All claims expressed in this article are solely those of the authors and do not necessarily represent those of their affiliated organizations, or those of the publisher, the editors and the reviewers. Any product that may be evaluated in this article, or claim that may be made by its manufacturer, is not guaranteed or endorsed by the publisher.
