# Supplementary material for: Soil Environments Influence Gut Prokaryotic Communities in the Larvae of the Invasive Japanese Beetle Popillia japonica Newman
Source: Front Microbiol. 2022 Apr 27;13:854513. doi: 10.3389/fmicb.2022.854513 (PMC9094118; doi:10.3389/fmicb.2022.854513)
Supplement: Supplementary file 1 [file Data_Sheet_1.pdf]

# *Supplementary Material*

## 1 Supplementary Figures and Tables

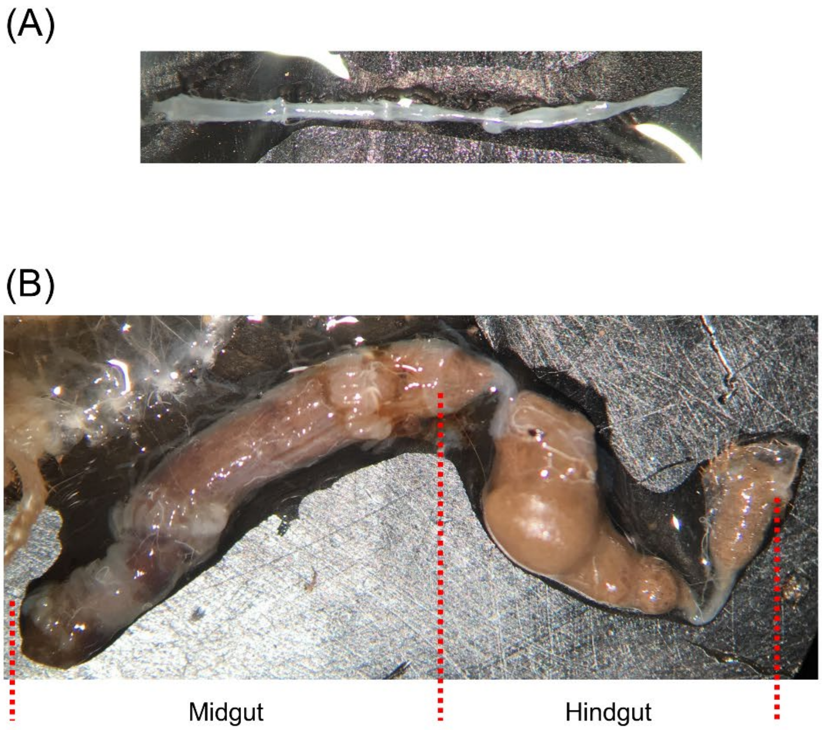


**Supplementary Figure 1.** Dissected alimentary tract from a neonate (A) and (B) a third instar Japanese beetle (*Popillia japonica* Newman) larvae. Alimentary tract from neonates were used entirely while alimentary tract from third instar larvae were divided into midgut (between the first set of gastric caeca after the head and the pyloric sphincter where the Malpighian tubules emerge) and hindgut (including ileum, colon, and rectum) sections.

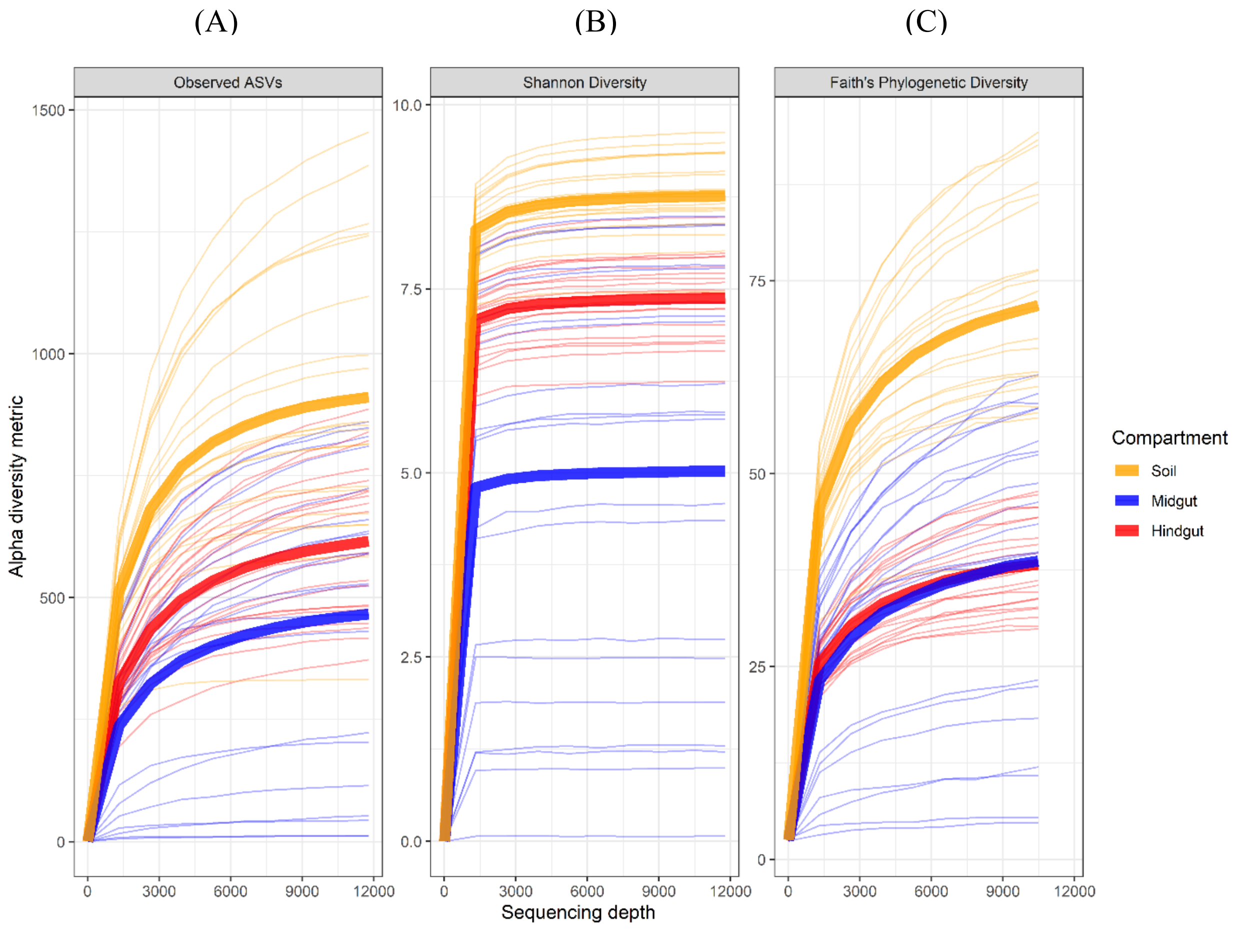


**Supplementary Figure 2.** Rarefaction curves for α-diversity metrics of prokaryotic communities in guts from third instar Japanese beetle (*Popillia japonica* Newman) larvae and associated soil, at a depth of 11,781 sequences. (A) Observed amplicon sequence variants (ASVs), (B) Shannon diversity, (C) Faith's phylogenetic diversity. Line color represents sample type (gut region or soil). Individual samples are represented by thin lines while average per sample type are represented by thick lines.

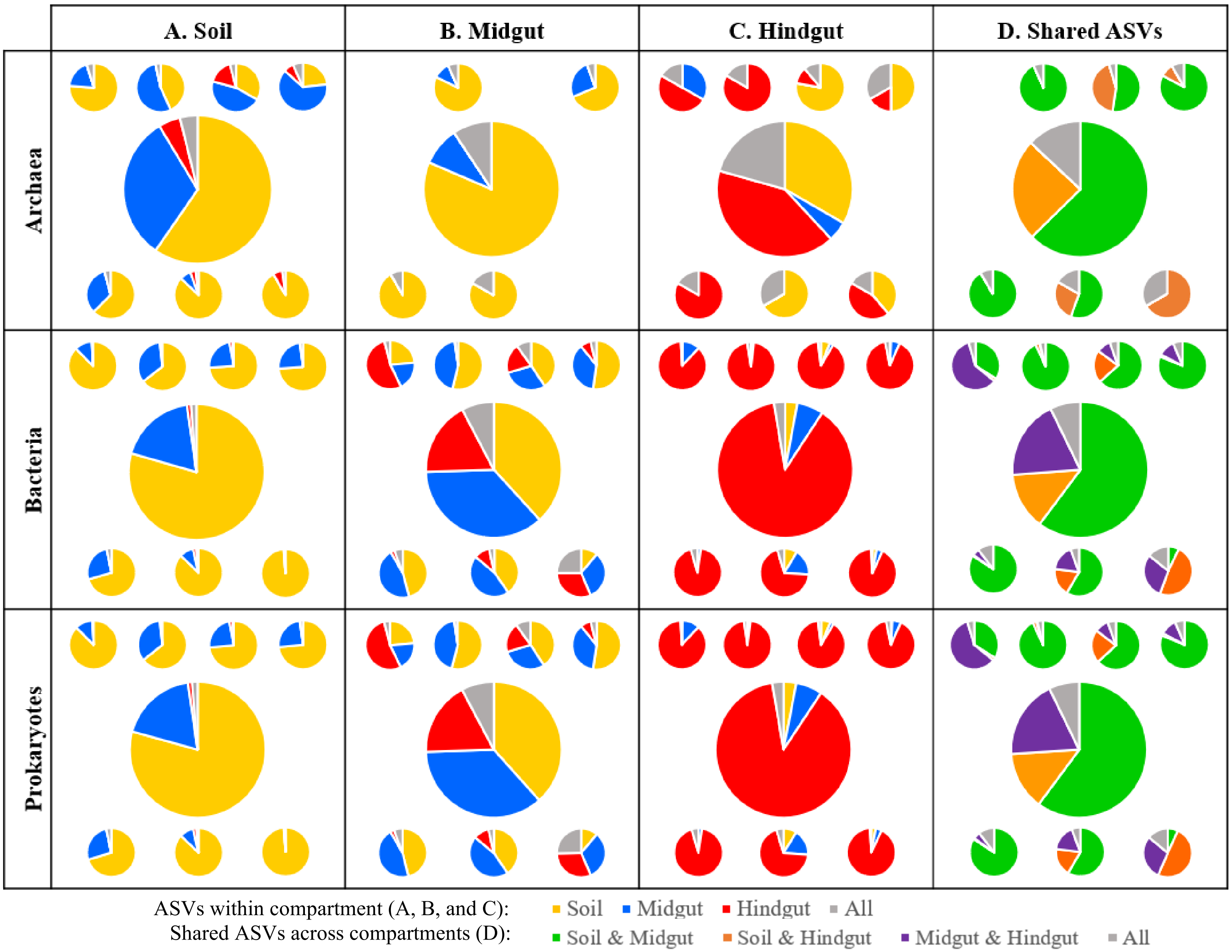
A. Soil
B. Midgut
C. Hindgut
D. Shared ASVs
Archaea
Bacteria
Prokaryotes
ASVs within compartment (A, B, and C):
Soil
Midgut
Hindgut
All
Shared ASVs across compartments (D):
Soil & Midgut
Soil & Hindgut
Midgut & Hindgut
All

**Supplementary Figure 3.** Distribution (percentage) of unique and shared observed amplicon sequence variants (ASVs) of the archaeal, bacterial and combined prokaryotic community within compartment: soil (A), and midgut (B) and hindgut (C) of third instar Japanese beetle (*Popillia japonica* Newman) larvae across locations. Shared ASVs (D) represents an overview of the distribution (percentage) of ASVs that are shared between two or three (all) compartments. Central larger chart represents the average ASVs across all locations. Smaller charts represent average ASVs per location as follows (left to right), in top row: Blackhawk, Culver, Janesville1, and Janesville2, and in bottom row: Nursery, Purdy, and TPAC. The cases where a smaller chart is not presented means that ASVs were not detected in that compartment/comparison at the location.

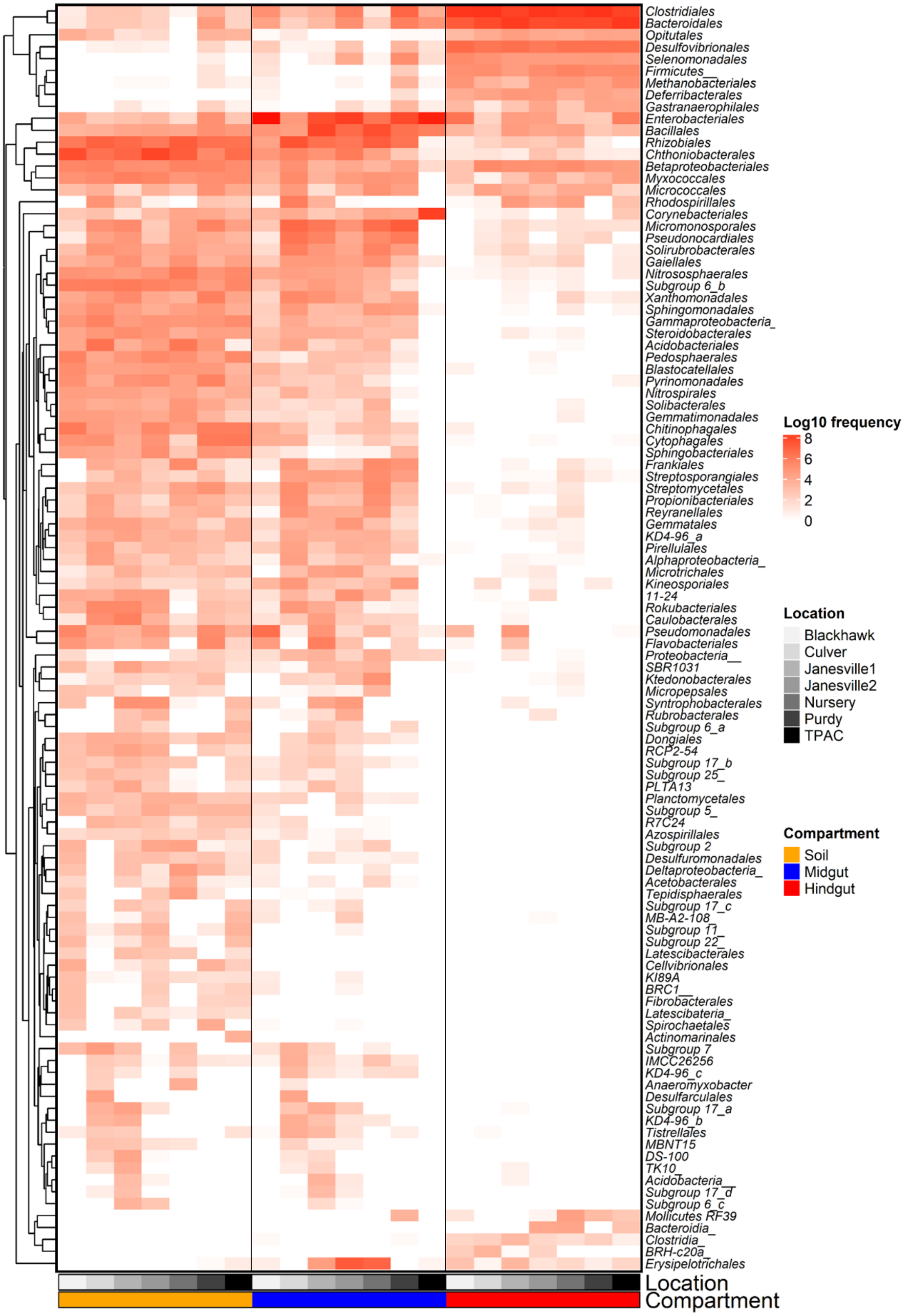
Clostridiales
Bacteroidales
Opitutales
Desulfovibrionales
Selenomonadales
Firmicutes__
Methanobacteriales
Deferribacterales
Gastranaerophilales
Enterobacteriales
Bacillales
Rhizobiales
Chthoniobacterales
Betaproteobacteriales
Myxococcales
Micrococcales
Rhodospirillales
Corynebacteriales
Micromonosporales
Pseudonocardiales
Solirubrobacterales
Gaiellales
Nitrososphaerales
Subgroup 6_b
Xanthomonadales
Sphingomonadales
Gammaproteobacteria_
Steroidobacterales
Acidobacteriales
Pedosphaerales
Blastocatellales
Pyrinomonadales
Nitrospirales
Solibacterales
Gemmatimonadales
Chitinophagales
Cytophagales
Sphingobacteriales
Frankiales
Streptosporangiales
Streptomycetales
Propionibacteriales
Reyranellales
Gemmatales
KD4-96_a
Pirellulales
Alphaproteobacteria_
Microtrichales
Kineosporiales
11-24
Rokubacteriales
Caulobacterales
Pseudomonadales
Flavobacteriales
Proteobacteria__
SBR1031
Ktedonobacterales
Micropepsales
Syntrophobacterales
Rubrobacterales
Subgroup 6_a
Dongiales
RCP2-54
Subgroup 17_b
Subgroup 25_
PLTA13
Planctomycetales
Subgroup 5_
R7C24
Azospirillales
Subgroup 2
Desulfuromonadales
Deltaproteobacteria_
Acetobacterales
Tepidisphaerales
Subgroup 17_c
MB-A2-108
Subgroup 11_
Subgroup 22_
Latescibacterales
Cellvibrionales
KI89A
BRC1__
Fibrobacterales
Latescibateria_
Spirochaetales
Actinomarinales
Subgroup 7
IMCC26256
KD4-96_c
Anaeromyxobacter
Desulfarculales
Subgroup 17_a
KD4-96_b
Tistrellales
MBNT15
DS-100
TK10
Acidobacteria__
Subgroup 17_d
Subgroup 6_c
Mollicutes RF39
Bacteroidia_
Clostridia_
BRH-c20a
Erysipelotrichales
Log10 frequency
8
6
4
2
0
Location
Blackhawk
Culver
Janesville1
Janesville2
Nursery
Purdy
TPAC
Compartment
Soil
Midgut
Hindgut
Location
Compartment

**Supplementary Figure 4.** Prokaryotic community based on V4 16S rRNA gene sequences at the order rank in gut (i.e., midgut, hindgut) from third instar larvae of the Japanese beetle (*Popillia japonica* Newman) and associated soil, at seven locations across Indiana and Wisconsin, USA. All taxa belong to *Bacteria* except to *Nitrososphaerales* and *Methanobacteriales* that belong to *Archaea*. Taxa with ASVs ≥0.016% in abundance were considered. The dendrogram is based on Euclidean dissimilarities between taxa. Average is presented, n=3 for each compartment at each location. For taxonomic affiliation at order rank refer to **Supplementary Table 3.**

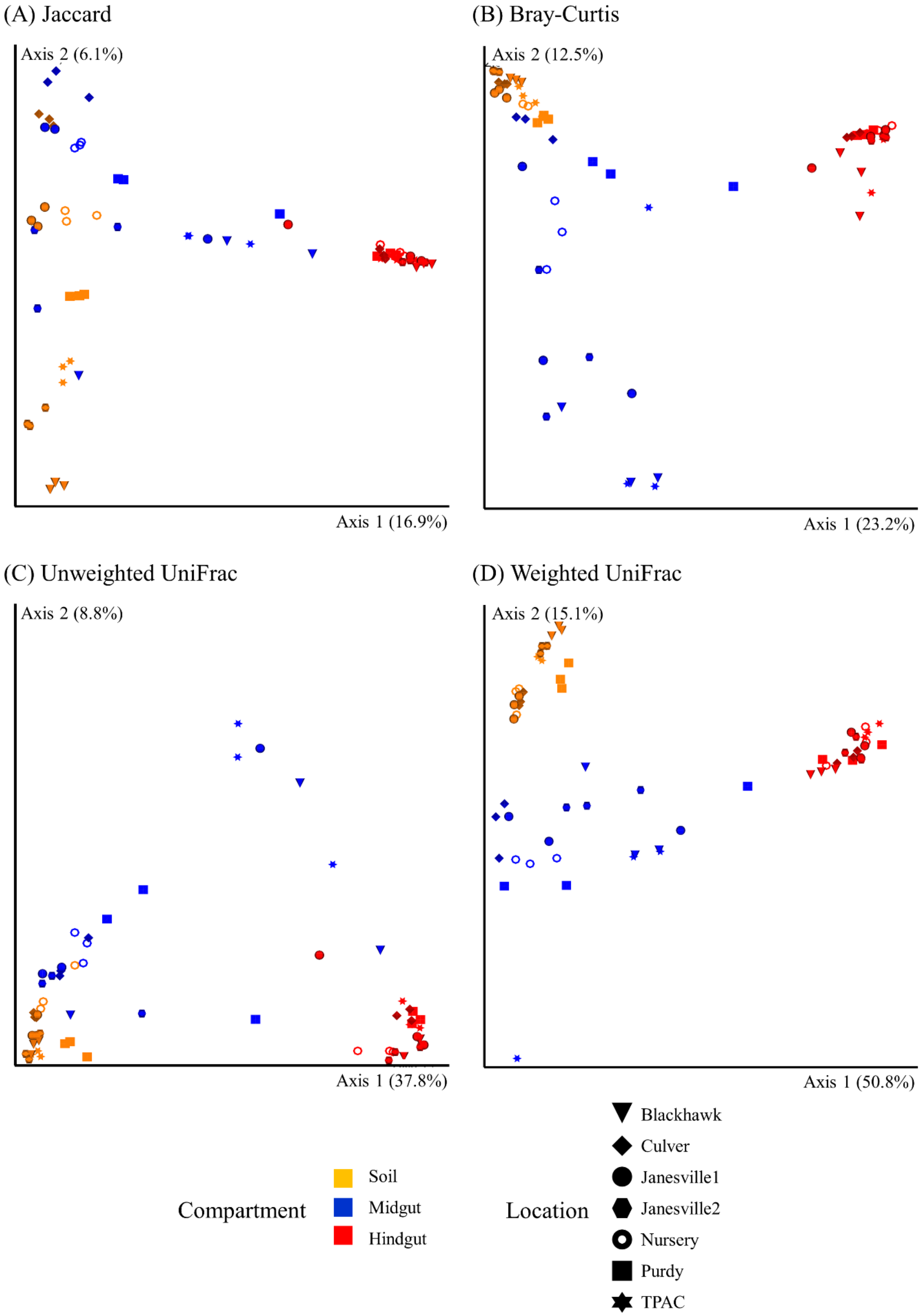

(A) Jaccard
Axis 2 (6.1%)
Axis 1 (16.9%)
(B) Bray-Curtis
Axis 2 (12.5%)
Axis 1 (23.2%)
(C) Unweighted UniFrac
Axis 2 (8.8%)
Axis 1 (37.8%)
(D) Weighted UniFrac
Axis 2 (15.1%)
Axis 1 (50.8%)
Compartment
Soil
Midgut
Hindgut
Location
Blackhawk
Culver
Janesville1
Janesville2
Nursery
Purdy
TPAC

**Supplementary Figure 5.** Microbial compositional profiles of prokaryotic communities in midgut and hindgut of third instar Japanese beetle (*Popillia japonica* Newman) larvae and associated soil visualized using the principal coordinate analysis (PCoA) based on (A) Jaccard-, (B) Bray-Curtis-, (C) Unweighted UniFrac-, and (D) Weighted UniFrac dissimilarity matrices. Data points represent individual samples where symbol color denotes compartment (i.e., midgut, hindgut, or soil) while symbol shape denotes location.

**Supplementary Table 1.** 16S rRNA gene reads recovered from Illumina MiSeq sequencing in the whole gut of first instar hatched under sterile conditions, and the midgut and hindgut regions of third instar Japanese beetle (*Popillia japonica* Newman) larvae and associated soil. For each compartment at each location, average ± SD are presented, n=3, except for first instar whole guts, where n=5.

| | Raw sequences | | | Filtered and denoised (DADA2) | | | Merged | | | Non-chimeric | | | Filtering based on >2, >p, nmnc* | | |
|---|---|---|---|---|---|---|---|---|---|---|---|---|---|---|---|
| **First instar** | 129,020 | ± | 60,928 | 123,366 | ± | 58,408 | 119,623 | ± | 55,525 | 114,977 | ± | 52,326 | 106,348 | ± | 49,771 |
| Janesville | 157,560 | ± | 45,011 | 150,458 | ± | 43,240 | 144,671 | ± | 39,708 | 138,876 | ± | 38,325 | 122,641 | ± | 41,543 |
| TPAC | 100,480 | ± | 65,499 | 96,275 | ± | 63,018 | 94,576 | ± | 61,577 | 91,078 | ± | 57,129 | 90,056 | ± | 56,429 |
| **Total** | **1,290,197** | | | **1,233,663** | | | **1,196,234** | | | **1,149,772** | | | **1,063,481** | | |
| **Soil** | **1,392,495** | **±** | **35,222** | **1,340,202** | **±** | **33,953** | **1,044,949** | **±** | **28,311** | **1,034,937** | **±** | **28,016** | **973,411** | **±** | **25,544** |
| Blackhawk | 134,338 | ± | 12,416 | 129,145 | ± | 11,925 | 100,949 | ± | 10,563 | 100,446 | ± | 10,463 | 96,187 | ± | 9,561 |
| Culver | 136,652 | ± | 7,996 | 131,393 | ± | 7,727 | 97,518 | ± | 5,804 | 96,471 | ± | 5,758 | 92,394 | ± | 5,446 |
| Janesville1 | 151,081 | ± | 17,118 | 144,982 | ± | 16,425 | 109,459 | ± | 13,143 | 108,075 | ± | 12,937 | 103,631 | ± | 12,148 |
| Janesville2 | 170,905 | ± | 28,866 | 164,409 | ± | 27,642 | 125,351 | ± | 22,537 | 124,344 | ± | 22,265 | 119,028 | ± | 20,731 |
| Nursery | 139,828 | ± | 27,826 | 134,643 | ± | 26,754 | 101,971 | ± | 21,310 | 101,003 | ± | 21,054 | 94,461 | ± | 19,203 |
| Purdy | 337,849 | ± | 40,889 | 326,078 | ± | 39,372 | 263,046 | ± | 33,750 | 260,442 | ± | 33,439 | 244,827 | ± | 30,424 |
| TPAC | 321,842 | ± | 21,079 | 309,552 | ± | 20,328 | 246,655 | ± | 17,036 | 244,156 | ± | 16,950 | 222,883 | ± | 14,828 |
| **Midgut** | **1,967,400** | **±** | **33,350** | **1,897,148** | **±** | **32,216** | **1,735,308** | **±** | **28,012** | **1,712,549** | **±** | **27,576** | **1,524,972** | **±** | **27,507** |
| Blackhawk | 306,525 | ± | 18,011 | 294,899 | ± | 17,219 | 280,535 | ± | 15,610 | 277,163 | ± | 15,982 | 262,166 | ± | 18,313 |
| Culver | 307,283 | ± | 32,489 | 296,168 | ± | 31,796 | 262,339 | ± | 28,234 | 258,648 | ± | 27,905 | 208,132 | ± | 23,098 |
| Janesville1 | 270,936 | ± | 40,640 | 261,758 | ± | 39,021 | 238,114 | ± | 32,697 | 234,161 | ± | 32,408 | 223,857 | ± | 29,879 |
| Janesville2 | 362,008 | ± | 29,827 | 349,464 | ± | 29,229 | 313,901 | ± | 26,535 | 309,146 | ± | 25,917 | 298,798 | ± | 25,653 |
| Nursery | 325,813 | ± | 7,981 | 313,611 | ± | 7,561 | 286,940 | ± | 5,376 | 282,613 | ± | 4,912 | 249,585 | ± | 2,991 |
| Purdy | 268,530 | ± | 33,353 | 259,521 | ± | 32,332 | 233,973 | ± | 26,976 | 232,081 | ± | 26,802 | 168,835 | ± | 31,325 |
| TPAC | 126,305 | ± | 18,477 | 121,727 | ± | 17,677 | 119,506 | ± | 16,961 | 118,737 | ± | 16,633 | 113,599 | ± | 18,325 |
| **Hindgut** | **2,030,261** | **±** | **33,947** | **1,952,179** | **±** | **32,718** | **1,771,213** | **±** | **30,454** | **1,760,927** | **±** | **30,214** | **1,711,363** | **±** | **29,260** |
| Blackhawk | 381,377 | ± | 19,597 | 366,796 | ± | 19,047 | 335,318 | ± | 18,482 | 333,820 | ± | 18,224 | 327,428 | ± | 17,838 |
| Culver | 210,721 | ± | 36,657 | 203,900 | ± | 36,047 | 186,581 | ± | 34,215 | 185,455 | ± | 33,969 | 178,363 | ± | 33,031 |
| Janesville1 | 308,466 | ± | 21,861 | 296,541 | ± | 21,520 | 271,187 | ± | 21,845 | 269,520 | ± | 21,308 | 260,446 | ± | 18,445 |
| Janesville2 | 333,289 | ± | 23,887 | 319,211 | ± | 23,051 | 284,944 | ± | 21,846 | 284,307 | ± | 21,824 | 276,631 | ± | 19,942 |
| Nursery | 370,986 | ± | 23,993 | 356,022 | ± | 23,098 | 321,129 | ± | 21,526 | 318,670 | ± | 21,554 | 307,611 | ± | 20,159 |
| Purdy | 151,478 | ± | 11,652 | 145,404 | ± | 11,107 | 128,729 | ± | 11,177 | 128,387 | ± | 11,202 | 124,472 | ± | 10,948 |
| TPAC | 273,944 | ± | 29,896 | 264,305 | ± | 29,090 | 243,325 | ± | 27,691 | 240,768 | ± | 27,110 | 236,412 | ± | 26,879 |
| **Total** | **5,390,156** | | | **5,189,529** | | | **4,551,470** | | | **4,508,413** | | | **4,209,746** | | |

*: >2: present in at least 2 samples; >p: assigned at least to the phylum rank of taxonomy classification; nmnc: were not classified as mitochondrial or chloroplast.

**Supplementary Table 2.** Number of observed amplicon sequence variants (ASVs) including taxonomic affiliation at different ranks, present in the prokaryotic community from midgut and hindgut of third instar Japanese beetle (*Popillia japonica* Newman) larvae and associated soil. Taxa for unrarefied and rarefied libraries are presented.

| | | | **Phylum** | | **Order** | | **Species** | | **ASVs** | |
|---|---|---|---|---|---|---|---|---|---|---|
| | | | ***Archaea*** | ***Bacteria*** | ***Archaea*** | ***Bacteria*** | ***Archaea*** | ***Bacteria*** | ***Archaea*** | ***Bacteria*** |
| No rarefaction | Total | ALL | 3 | 34 | 4 | 355 | 15 | 1,885 | 37 | 8,381 |
| | | Soil | 3 | 34 | 4 | 338 | 13 | 1,616 | 34 | 5,385 |
| | | Midgut | 2 | 27 | 2 | 248 | 9 | 1,349 | 26 | 4,274 |
| | | Hindgut | 2 | 17 | 2 | 99 | 6 | 437 | 11 | 2,762 |
| | Unique | Soil | 1 | 7 | 2 | 105 | 5 | 471 | 10 | 2,530 |
| | | Midgut | 0 | 0 | 0 | 11 | 1 | 104 | 1 | 653 |
| | | Hindgut | 0 | 0 | 0 | 1 | 1 | 53 | 1 | 1,513 |
| | Shared | ALL | 2 | 17 | 2 | 92 | 5 | 260 | 9 | 355 |
| | | Soil & Midgut | 0 | 10 | 0 | 140 | 3 | 873 | 15 | 2,436 |
| | | Soil & Hindgut | 0 | 0 | 0 | 1 | 0 | 12 | 0 | 64 |
| | | Midgut & Hindgut | 0 | 0 | 0 | 5 | 0 | 112 | 1 | 830 |
| Rarefaction at 11,781 | Total | ALL | 3 | 34 | 4 | 355 | 15 | 1,877 | 37 | 8,333 |
| | | Soil | 3 | 33 | 4 | 334 | 13 | 1,589 | 33 | 5,294 |
| | | Midgut | 2 | 24 | 2 | 236 | 9 | 1,293 | 25 | 4,026 |
| | | Hindgut | 2 | 15 | 2 | 87 | 5 | 400 | 9 | 2,676 |
| | Unique | Soil | 1 | 10 | 2 | 117 | 5 | 516 | 11 | 2,678 |
| | | Midgut | 0 | 0 | 0 | 14 | 1 | 118 | 2 | 716 |
| | | Hindgut | 0 | 0 | 0 | 2 | 1 | 58 | 1 | 1,580 |
| | Shared | ALL | 2 | 14 | 2 | 80 | 4 | 220 | 7 | 304 |
| | | Soil & Midgut | 0 | 9 | 0 | 137 | 4 | 843 | 15 | 2,263 |
| | | Soil & Hindgut | 0 | 0 | 0 | 0 | 0 | 10 | 0 | 49 |
| | | Midgut & Hindgut | 0 | 1 | 0 | 5 | 0 | 112 | 1 | 743 |
| Rarefaction coverage (%) | | ALL | 100.0 | 100.0 | 100.0 | 100.0 | 100.0 | 99.6 | 100.0 | 99.4 |
| | | Soil | 100.0 | 97.1 | 100.0 | 98.8 | 100.0 | 98.3 | 97.1 | 98.3 |
| | | Midgut | 100.0 | 88.9 | 100.0 | 95.2 | 100.0 | 95.8 | 96.2 | 94.2 |
| | | Hindgut | 100.0 | 88.2 | 100.0 | 87.9 | 83.3 | 91.5 | 81.8 | 96.9 |

**Supplementary Table 3.** Taxonomic affiliation (SILVA v132) of prokaryotic community (up to order rank when available) in gut (i.e., midgut, hindgut) from third instar larvae of the Japanese beetle (*Popillia japonica* Newman) and associated soil.

| Label in heatmap | Kingdom | Phylum | Class | Order |
|---|---|---|---|---|
| *11-24* | *Bacteria* | *Acidobacteria* | *Blastocatellia (Subgroup 4)* | *11-24* |
| *Acetobacterales* | *Bacteria* | *Proteobacteria* | *Alphaproteobacteria* | *Acetobacterales* |
| *Acidobacteria__* | *Bacteria* | *Actinobacteria* | __ | __ |
| *Acidobacteriales* | *Bacteria* | *Acidobacteria* | *Acidobacteriia* | *Acidobacteriales* |
| *Actinomarinales* | *Bacteria* | *Actinobacteria* | *Acidimicrobiia* | *Actinomarinales* |
| *Alphaproteobacteria_* | *Bacteria* | *Proteobacteria* | *Alphaproteobacteria* | *uncultured* |
| *Anaeromyxobacter* | *Bacteria* | *Acidobacteria* | *Subgroup 6* | *uncultured Anaeromyxobacter sp.* |
| *Azospirillales* | *Bacteria* | *Proteobacteria* | *Alphaproteobacteria* | *Azospirillales* |
| *Bacillales* | *Bacteria* | *Firmicutes* | *Bacilli* | *Bacillales* |
| *Bacteroidales* | *Bacteria* | *Bacteroidetes* | *Bacteroidia* | *Bacteroidales* |
| *Bacteroidia_* | *Bacteria* | *Bacteroidetes* | *Bacteroidia* | __ |
| *Betaproteobacteriales* | *Bacteria* | *Proteobacteria* | *Gammaproteobacteria* | *Betaproteobacteriales* |
| *Blastocatellales* | *Bacteria* | *Acidobacteria* | *Blastocatellia (Subgroup 4)* | *Blastocatellales* |
| *BRC1__* | *Bacteria* | *BRC1* | *uncultured Acidobacteria bacterium* | *uncultured Acidobacteria bacterium* |
| *BRH-c20a_* | *Bacteria* | *Firmicutes* | *BRH-c20a* | *uncultured bacterium* |
| *Caulobacterales* | *Bacteria* | *Proteobacteria* | *Alphaproteobacteria* | *Caulobacterales* |
| *Cellvibrionales* | *Bacteria* | *Proteobacteria* | *Gammaproteobacteria* | *Cellvibrionales* |
| *Chitinophagales* | *Bacteria* | *Bacteroidetes* | *Bacteroidia* | *Chitinophagales* |
| *Chthoniobacterales* | *Bacteria* | *Verrucomicrobia* | *Verrucomicrobiae* | *Chthoniobacterales* |
| *Clostridia_* | *Bacteria* | *Firmicutes* | *Clostridia* | __ |
| *Clostridiales* | *Bacteria* | *Firmicutes* | *Clostridia* | *Clostridiales* |
| *Corynebacteriales* | *Bacteria* | *Actinobacteria* | *Actinobacteria* | *Corynebacteriales* |
| *Cytophagales* | *Bacteria* | *Bacteroidetes* | *Bacteroidia* | *Cytophagales* |
| *Deferribacterales* | *Bacteria* | *Deferribacteres* | *Deferribacteres* | *Deferribacterales* |
| *Deltaproteobacteria_* | *Bacteria* | *Proteobacteria* | *Deltaproteobacteria* | __ |
| *Desulfarculales* | *Bacteria* | *Proteobacteria* | *Deltaproteobacteria* | *Desulfarculales* |
| *Desulfovibrionales* | *Bacteria* | *Proteobacteria* | *Deltaproteobacteria* | *Desulfovibrionales* |
| *Desulfuromonadales* | *Bacteria* | *Proteobacteria* | *Deltaproteobacteria* | *Desulfuromonadales* |
| *Dongiales* | *Bacteria* | *Proteobacteria* | *Alphaproteobacteria* | *Dongiales* |
| *DS-100* | *Bacteria* | *Acidobacteria* | *Blastocatellia (Subgroup 4)* | *DS-100* |
| *Enterobacteriales* | *Bacteria* | *Proteobacteria* | *Gammaproteobacteria* | *Enterobacteriales* |
| *Erysipelotrichales* | *Bacteria* | *Firmicutes* | *Erysipelotrichia* | *Erysipelotrichales* |
| *Fibrobacterales* | *Bacteria* | *Fibrobacteres* | *Fibrobacteria* | *Fibrobacterales* |
| *Firmicutes__* | *Bacteria* | *Firmicutes* | __ | __ |
| *Flavobacteriales* | *Bacteria* | *Bacteroidetes* | *Bacteroidia* | *Flavobacteriales* |
| *Frankiales* | *Bacteria* | *Actinobacteria* | *Actinobacteria* | *Frankiales* |
| *Gaiellales* | *Bacteria* | *Actinobacteria* | *Thermoleophilia* | *Gaiellales* |
| *Gammaproteobacteria_* | *Bacteria* | *Proteobacteria* | *Gammaproteobacteria* | *Gammaproteobacteria Incertae Sedis* |
| *Gastranaerophilales* | *Bacteria* | *Cyanobacteria* | *Melainabacteria* | *Gastranaerophilales* |
| *Gemmatales* | *Bacteria* | *Planctomycetes* | *Planctomycetacia* | *Gemmatales* |
| *Gemmatimonadales* | *Bacteria* | *Gemmatimonadetes* | *Gemmatimonadetes* | *Gemmatimonadales* |
| *IMCC26256* | *Bacteria* | *Actinobacteria* | *Acidimicrobiia* | *IMCC26256* |

**Supplementary Table 3**. *Continued*

| **Label in heatmap** | **Kingdom** | **Phylum** | **Class** | **Order** |
|---|---|---|---|---|
| *KD4-96_a* | *Bacteria* | *Chloroflexi* | *KD4-96* | __ |
| *KD4-96_b* | *Bacteria* | *Chloroflexi* | *KD4-96* | *uncultured Chloroflexi bacterium* |
| *KD4-96_c* | *Bacteria* | *Chloroflexi* | *KD4-96* | *uncultured bacterium* |
| *KI89A* | *Bacteria* | *Proteobacteria* | *Gammaproteobacteria* | *KI89A clade* |
| *Kineosporiales* | *Bacteria* | *Actinobacteria* | *Actinobacteria* | *Kineosporiales* |
| *Ktedonobacterales* | *Bacteria* | *Chloroflexi* | *Ktedonobacteria* | *Ktedonobacterales* |
| *Latescibacterales* | *Bacteria* | *Latescibacteria* | *Latescibacteria* | *Latescibacterales* |
| *Latescibateria_* | *Bacteria* | *Latescibacteria* | *metagenome* | *metagenome* |
| *MB-A2-108_* | *Bacteria* | *Actinobacteria* | *MB-A2-108* | *metagenome* |
| *MBNT15* | *Bacteria* | *Proteobacteria* | *Deltaproteobacteria* | *MBNT15* |
| *Methanobacteriales* | *Archaea* | *Euryarchaeota* | *Methanobacteria* | *Methanobacteriales* |
| *Micrococcales* | *Bacteria* | *Actinobacteria* | *Actinobacteria* | *Micrococcales* |
| *Micromonosporales* | *Bacteria* | *Actinobacteria* | *Actinobacteria* | *Micromonosporales* |
| *Micropepsales* | *Bacteria* | *Proteobacteria* | *Alphaproteobacteria* | *Micropepsales* |
| *Microtrichales* | *Bacteria* | *Actinobacteria* | *Acidimicrobiia* | *Microtrichales* |
| *Mollicutes RF39* | *Bacteria* | *Tenericutes* | *Mollicutes* | *Mollicutes RF39* |
| *Myxococcales* | *Bacteria* | *Proteobacteria* | *Deltaproteobacteria* | *Myxococcales* |
| *Nitrososphaerales* | *Archaea* | *Thaumarchaeota* | *Nitrososphaeria* | *Nitrososphaerales* |
| *Nitrospirales* | *Bacteria* | *Nitrospirae* | *Nitrospira* | *Nitrospirales* |
| *Opitutales* | *Bacteria* | *Verrucomicrobia* | *Verrucomicrobiae* | *Opitutales* |
| *Pedosphaerales* | *Bacteria* | *Verrucomicrobia* | *Verrucomicrobiae* | *Pedosphaerales* |
| *Pirellulales* | *Bacteria* | *Planctomycetes* | *Planctomycetacia* | *Pirellulales* |
| *Planctomycetales* | *Bacteria* | *Planctomycetes* | *Planctomycetacia* | *Planctomycetales* |
| *PLTA13* | *Bacteria* | *Proteobacteria* | *Gammaproteobacteria* | *PLTA13* |
| *Propionibacteriales* | *Bacteria* | *Actinobacteria* | *Actinobacteria* | *Propionibacteriales* |
| *Proteobacteria__* | *Bacteria* | *Proteobacteria* | __ | __ |
| *Pseudomonadales* | *Bacteria* | *Proteobacteria* | *Gammaproteobacteria* | *Pseudomonadales* |
| *Pseudonocardiales* | *Bacteria* | *Actinobacteria* | *Actinobacteria* | *Pseudonocardiales* |
| *Pyrinomonadales* | *Bacteria* | *Acidobacteria* | *Blastocatellia (Subgroup 4)* | *Pyrinomonadales* |
| *R7C24* | *Bacteria* | *Proteobacteria* | *Gammaproteobacteria* | *R7C24* |
| *RCP2-54* | *Bacteria* | *Proteobacteria* | *Deltaproteobacteria* | *RCP2-54* |
| *Reyranellales* | *Bacteria* | *Proteobacteria* | *Alphaproteobacteria* | *Reyranellales* |
| *Rhizobiales* | *Bacteria* | *Proteobacteria* | *Alphaproteobacteria* | *Rhizobiales* |
| *Rhodospirillales* | *Bacteria* | *Proteobacteria* | *Alphaproteobacteria* | *Rhodospirillales* |
| *Rokubacteriales* | *Bacteria* | *Rokubacteria* | *NC10* | *Rokubacteriales* |
| *Rubrobacterales* | *Bacteria* | *Actinobacteria* | *Rubrobacteria* | *Rubrobacterales* |
| *SBR1031* | *Bacteria* | *Chloroflexi* | *Anaerolineae* | *SBR1031* |
| *Selenomonadales* | *Bacteria* | *Firmicutes* | *Negativicutes* | *Selenomonadales* |
| *Solibacterales* | *Bacteria* | *Acidobacteria* | *Acidobacteriia* | *Solibacterales* |
| *Solirubrobacterales* | *Bacteria* | *Actinobacteria* | *Thermoleophilia* | *Solirubrobacterales* |
| *Sphingobacteriales* | *Bacteria* | *Bacteroidetes* | *Bacteroidia* | *Sphingobacteriales* |
| *Sphingomonadales* | *Bacteria* | *Proteobacteria* | *Alphaproteobacteria* | *Sphingomonadales* |
| *Spirochaetales* | *Bacteria* | *Spirochaetes* | *Spirochaetia* | *Spirochaetales* |
| *Steroidobacterales* | *Bacteria* | *Proteobacteria* | *Gammaproteobacteria* | *Steroidobacterales* |
| *Streptomycetales* | *Bacteria* | *Actinobacteria* | *Actinobacteria* | *Streptomycetales* |
| *Streptosporangiales* | *Bacteria* | *Actinobacteria* | *Actinobacteria* | *Streptosporangiales* |

**Supplementary Table 3**. *Continued*

| Label in heatmap | Kingdom | Phylum | Class | Order |
|---|---|---|---|---|
| *Subgroup 11_* | *Bacteria* | *Acidobacteria* | *Subgroup 11* | *uncultured bacterium* |
| *Subgroup 17_a* | *Bacteria* | *Acidobacteria* | *Subgroup 17* | __ |
| *Subgroup 17_b* | *Bacteria* | *Acidobacteria* | *Subgroup 17* | *uncultured Acidobacteriales bacterium* |
| *Subgroup 17_c* | *Bacteria* | *Acidobacteria* | *Subgroup 17* | *uncultured Acidobacteria bacterium* |
| *Subgroup 17_d* | *Bacteria* | *Acidobacteria* | *Subgroup 17* | *uncultured bacterium* |
| *Subgroup 2* | *Bacteria* | *Acidobacteria* | *Acidobacteriia* | *Subgroup 2* |
| *Subgroup 22_* | *Bacteria* | *Acidobacteria* | *Subgroup 22* | *uncultured Holophaga sp.* |
| *Subgroup 25_* | *Bacteria* | *Acidobacteria* | *Subgroup 25* | *uncultured soil bacterium* |
| *Subgroup 5_* | *Bacteria* | *Acidobacteria* | *Subgroup 5* | __ |
| *Subgroup 6_a* | *Bacteria* | *Acidobacteria* | *Subgroup 6* | *Unknown Order* |
| *Subgroup 6_b* | *Bacteria* | *Acidobacteria* | *Subgroup 6* | __ |
| *Subgroup 6_c* | *Bacteria* | *Acidobacteria* | *Subgroup 6* | *uncultured Acidobacterium sp.* |
| *Subgroup 7* | *Bacteria* | *Acidobacteria* | *Holophagae* | *Subgroup 7* |
| *Syntrophobacterales* | *Bacteria* | *Proteobacteria* | *Deltaproteobacteria* | *Syntrophobacterales* |
| *Tepidisphaerales* | *Bacteria* | *Planctomycetes* | *Phycisphaerae* | *Tepidisphaerales* |
| *Tistrellales* | *Bacteria* | *Proteobacteria* | *Alphaproteobacteria* | *Tistrellales* |
| *TK10_* | *Bacteria* | *Chloroflexi* | *TK10* | __ |
| *Xanthomonadales* | *Bacteria* | *Proteobacteria* | *Gammaproteobacteria* | *Xanthomonadales* |

**Supplementary Table 4.** Influence of compartment (i.e., midgut, hindgut, or soil), location, and their interaction on variation in α-diversity of prokaryotic communities in the alimentary tract of third instar Japanese beetle (*Popillia japonica* Newman) larvae and associated soil. F-statistics and *p*-values for Aligned Rank Transform (ART) nonparametric factorial ANOVA.

| **Factor** | ***df*[1]** | **Observed ASVs[2]** | | **Evenness** | | **Shannon Diversity** | | **Faith's PD[3]** | |
|---|---|---|---|---|---|---|---|---|---|
| | | F | *p*-value | F | *p*-value | F | *p*-value | F | *p*-value |
| Compartment | 2 | 37.9 | < 0.001 | 106.7 | < 0.001 | 111.0 | < 0.001 | 38.6 | < 0.001 |
| Location | 6 | 0.9 | 0.505 | 7.4 | < 0.001 | 8.1 | < 0.001 | 2.2 | 0.061 |
| Compartment × Location | 12 | 4.8 | < 0.001 | 5.7 | < 0.001 | 7.7 | < 0.001 | 5.2 | < 0.001 |
| Error | 42 | | | | | | | | |

[1]*df*= degrees of freedom; [2]ASVs= Amplicon sequence variants (Richness); [3]Faith's PD= Faith's phylogenetic distance

**Supplementary Table 5.** Pairwise comparisons between compartments on prokaryotic α-diversity in the alimentary tract of third instar Japanese beetle (*Popillia japonica* Newman) larvae and associated soil. Across-location comparisons emphasize relationships between compartments by comparing the slopes between compartments across locations using contrasts for the Aligned Rank Transform (ART) nonparametric ANOVA to generate differences of differences (entries with different letters are significantly different at α=0.05 using Tukey-corrected for multiple comparisons *p*-values). Within-location comparisons emphasize relationships between compartments (soil *versus* midgut, soil *versus* hindgut, and midgut *versus* hindgut) at each location independently by comparing mean α-diversity values using Kruskal-Wallis nonparametric ANOVA (> and < indicate statistical significance and the relative trajectory of those differences at α=0.05). n=3 for each compartment at each location.

| | | **Observed ASVs[1]** | | **Evenness** | | **Shannon Diversity** | | **Faith's PD[2]** | |
|---|---|---|---|---|---|---|---|---|---|
| | **Location** | **Across locations** | **Within location** | **Across locations** | **Within location** | **Across locations** | **Within location** | **Across locations** | **Within location** |
| **Soil *vs.* Midgut** | Blackhawk | ab | Soil > Midgut | a | Soil > Midgut | a | Soil > Midgut | ab | Soil > Midgut |
| | Culver | a | ND | b | Soil > Midgut | b | Soil > Midgut | c | ND |
| | Janesville1 | ab | ND | c | Soil > Midgut | c | Soil > Midgut | abc | ND |
| | Janesville2 | a | ND | c | Soil > Midgut | c | Soil > Midgut | ac | Soil > Midgut |
| | Nursery | a | ND | bc | Soil > Midgut | bc | Soil > Midgut | c | ND |
| | Purdy | b | Soil > Midgut | bc | Soil > Midgut | c | Soil > Midgut | b | Soil > Midgut |
| | TPAC | c | Soil > Midgut | a | Soil > Midgut | a | Soil > Midgut | d | Soil > Midgut |
| **Soil *vs.* Hindgut** | Blackhawk | a | ND | a | Soil > Hindgut | ab | ND | ab | Soil > Hindgut |
| | Culver | a | Soil > Hindgut | a | Soil > Hindgut | ab | Soil > Hindgut | ac | Soil > Hindgut |
| | Janesville1 | a | ND | a | Soil > Hindgut | ab | Soil > Hindgut | ac | Soil > Hindgut |
| | Janesville2 | a | ND | a | Soil > Hindgut | a | Soil > Hindgut | a | Soil > Hindgut |
| | Nursery | a | ND | a | Soil > Hindgut | ab | ND | b | ND |
| | Purdy | b | Soil > Hindgut | a | Soil > Hindgut | ab | Soil > Hindgut | d | Soil > Hindgut |
| | TPAC | b | Soil > Hindgut | a | Soil > Hindgut | b | Soil > Hindgut | cd | Soil > Hindgut |
| **Midgut *vs.* Hindgut** | Blackhawk | a | Midgut < Hindgut | a | Midgut < Hindgut | a | Midgut < Hindgut | ab | ND |
| | Culver | b | ND | b | Midgut > Hindgut | b | Midgut > Hindgut | c | Midgut > Hindgut |
| | Janesville1 | ab | ND | bcd | ND | c | ND | acd | ND |
| | Janesville2 | ab | ND | ce | Midgut < Hindgut | c | Midgut < Hindgut | acd | Midgut > Hindgut |
| | Nursery | ab | Midgut < Hindgut | bd | ND | bc | Midgut < Hindgut | abd | ND |
| | Purdy | b | ND | cd | ND | bc | ND | cd | ND |
| | TPAC | a | Midgut < Hindgut | ae | Midgut < Hindgut | a | Midgut < Hindgut | b | Midgut < Hindgut |

[1]ASVs= Amplicon sequence variants (Richness); [2]Faith's PD=Faith's phylogenetic distance; ND= no significant difference between samples.

**Supplementary Table 6.** Pairwise comparisons within compartment on prokaryotic α-diversity in the alimentary tract of third instar Japanese beetle (*Popillia japonica* Newman) larvae and associated soil. Comparisons emphasize differences within a single compartment (soil, midgut, or hindgut) across locations by comparing mean values using a one-way ANOVA and Tukey's-HSD post hoc (entries with different letters are statistically significant at α=0.05). n=3 for each compartment at each location.

| | **Location** | **Observed ASVs[1]** | **Evenness** | **Shannon Diversity** | **Faith's PD[2]** |
|---|---|---|---|---|---|
| **Soil** | ANOVA: $F_{6,14}$, *p*-value | 5.030, 0.006 | 18.46, <0.001 | 5.795, 0.003 | 5.613, 0.004 |
| | Blackhawk | bc | bc | bc | abc |
| | Culver | bc | a | abc | bc |
| | Janesville1 | abc | a | abc | abc |
| | Janesville2 | abc | c | bc | abc |
| | Nursery | c | ab | c | c |
| | Purdy | ab | a | ab | ab |
| | TPAC | a | a | a | a |
| **Midgut** | ANOVA: $F_{6,14}$, *p*-value | 2.592, 0.067 | 4.882, 0.007 | 5.722, 0.003 | 2.552, 0.070 |
| | Blackhawk | a | b | bc | a |
| | Culver | a | a | a | a |
| | Janesville1 | a | ab | abc | a |
| | Janesville2 | a | ab | abc | a |
| | Nursery | a | ab | ab | a |
| | Purdy | a | ab | abc | a |
| | TPAC | a | b | c | a |
| **Hindgut** | ANOVA: $F_{6,14}$, *p*-value | 6.955, 0.001 | 3.245, 0.033 | 3.644, 0.022 | 11.470, <0.001 |
| | Blackhawk | a | a | a | abc |
| | Culver | c | a | ab | d |
| | Janesville1 | abc | a | ab | bcd |
| | Janesville2 | ab | a | ab | ab |
| | Nursery | ab | a | ab | a |
| | Purdy | c | a | ab | d |
| | TPAC | bc | a | b | cd |

[1]ASVs= Amplicon sequence variants (Richness); [2]Faith's PD=Faith's phylogenetic distance

**Supplementary Table 7.** Influence of compartment (midgut, hindgut, soil), location, and their interaction on variation in β-diversity of prokaryotic communities in the alimentary tract of third instar Japanese beetle (*Popillia japonica* Newman) larvae and associated soil. Pseudo-F statistics, $R^2$ and *p*-values for factorial permutational analysis of variance (PERMANOVA, Adonis) model used 999 permutations.

| | | Jaccard | | | Bray-Curtis | | | Unweighted UniFrac | | | Weighted UniFrac | | | DEICODE | | |
|---|---|---|---|---|---|---|---|---|---|---|---|---|---|---|---|---|
| **Term** | ***df*[1]** | **pseudo-F** | ***p*-value** | **$R^2$** | **pseudo-F** | ***p*-value** | **$R^2$** | **pseudo-F** | ***p*-value** | **$R^2$** | **pseudo-F** | ***p*-value** | **$R^2$** | **pseudo-F** | ***p*-value** | **$R^2$** |
| Compartment | 2 | 10.9 | 0.001 | 0.191 | 27.1 | 0.001 | 0.313 | 30.2 | 0.001 | 0.389 | 76.1 | 0.001 | 0.599 | 174.2 | 0.001 | 0.654 |
| Location | 6 | 3.6 | 0.001 | 0.187 | 5.5 | 0.001 | 0.189 | 3.7 | 0.001 | 0.144 | 4.2 | 0.001 | 0.099 | 9.0 | 0.001 | 0.101 |
| Compartment × Location | 12 | 2.4 | 0.001 | 0.254 | 3.7 | 0.001 | 0.257 | 2.5 | 0.001 | 0.196 | 2.9 | 0.001 | 0.138 | 7.4 | 0.001 | 0.166 |
| Residuals | 42 | | | 0.368 | | | 0.242 | | | 0.271 | | | 0.165 | | | 0.079 |

[1]*df*=degrees of freedom

**Supplementary Table 8.** Homogeneity of dispersion among compartments (midgut, hindgut, soil) and location of prokaryotic communities in the alimentary tract of third instar Japanese beetle (*Popillia japonica* Newman) larvae and associated soil. Notice that the test of dispersions was run separately and independently for the two factors. F-statistics and *p*-values for permutational analysis of multivariate dispersion (PERMDISP) models used 999 permutations.

| | | | Jaccard | | Bray-Curtis | | Unweighted UniFrac | | Weighted UniFrac | | DEICODE | |
|---|---|---|---|---|---|---|---|---|---|---|---|---|
| **Term** | **n** | **Groups** | **F-value** | ***p*-value** | **F-value** | ***p*-value** | **F-value** | ***p*-value** | **F-value** | ***p*-value** | **F-value** | ***p*-value** |
| **Compartment** | **63** | **3** | **30.0** | **0.001** | 12.0 | **0.001** | **36.1** | **0.001** | **22.8** | **0.001** | **8.3** | **0.001** |
| *Soil vs. Midgut* | 42 | 2 | 11.4 | 0.007 | 2.6 | 0.073 | 20.8 | 0.001 | 22.3 | 0.002 | 0.2 | 0.596 |
| *Soil vs. Hindgut* | 42 | 2 | 25.7 | 0.002 | 18.4 | 0.002 | 26.4 | 0.001 | 1.4 | 0.003 | 14.3 | 0.002 |
| *Midgut vs. Hindgut* | 42 | 2 | 47.5 | 0.002 | 18.8 | 0.002 | 53.5 | 0.001 | 26.5 | 0.002 | 13.9 | 0.002 |
| **Location** | **63** | **7** | **0.4** | **0.627** | **0.4** | **0.501** | **0.5** | **0.366** | **0.6** | **0.403** | **0.1** | **0.977** |

**Supplementary Table 9.** Selection of key prokaryotic taxa for pairwise analysis of log-ratios across compartments: midgut and hindgut from third instar larvae of the Japanese beetle (*Popillia japonica* Newman), and associated soil. Taxa are presented in order of criteria for consideration, including most prevalent and/or top taxa driving most of the difference among compartments. Number of amplicon sequence variants (ASVs) assigned to each taxon, as well as their prevalence in samples (21 samples= 100%) and locations (7 locations= 100%) per compartment are presented.

| Taxa | Criteria for consideration* | # ASVs | Soil | | Midgut | | Hindgut | | Key taxa for log ratio analysis** |
|---|---|---|---|---|---|---|---|---|---|
| | | | Sample prevalence (%) | Location prevalence (%) | Sample prevalence (%) | Location prevalence (%) | Sample prevalence (%) | Location prevalence (%) | |
| *Rhizobiales* | *i*. s, m; *ii.* | 234 | 100 | 100 | 76 | 100 | 100 | 100 | Yes |
| *Chthoniobacterales* | *i*. s; *ii*. | 142 | 100 | 100 | 95 | 100 | 100 | 100 | Yes |
| *Betaproteobacteriales* | *i*, s. | 222 | 100 | 100 | 90 | 100 | 100 | 100 | Yes |
| *Enterobacteriales* | *i*. m; *ii.* | 10 | 95 | 100 | 100 | 100 | 100 | 100 | Yes |
| *Bacillales* | *i*. m; *ii.* | 61 | 100 | 100 | 100 | 100 | 100 | 100 | Yes |
| *Clostridiales* | *i*. h; *ii.* | 2,106 | 95 | 100 | 95 | 100 | 100 | 100 | Yes |
| *Bacteroidales* | *i*. h; *ii.* | 47 | 81 | 86 | 95 | 100 | 100 | 100 | Yes |
| *Desulfovibrionales* | *i*. h; *ii.* | 22 | 43 | 71 | 71 | 100 | 100 | 100 | Yes |
| *Chitinophagales* | *ii* | 263 | 95 | 100 | 81 | 100 | <u>14</u> | <u>29</u> | No |
| *Erysipelotrichales* | *ii* | 24 | <u>29</u> | <u>29</u> | 57 | 100 | 100 | 100 | No |
| *Steroidobacterales* | *ii* | 23 | 100 | 100 | 76 | 86 | <u>29</u> | 71 | No |

[*] ***Criteria for consideration:***

*i*. The three most prevalent orders in the prokaryotic core microbiota of each compartment (**Figure 3**), s=soil, m=midgut, h=hindgut;

*ii*. Orders of top 20 ASVs driving most of the variation among compartments in the ordination space (**Figure 4**);

[**] ***Criteria for selection:*** Sample and location prevalence are both ≥33% in all compartments (the log-ratio method is sensitive to sparsity).

**Supplementary Table 10.** Pairwise analysis of prokaryotic taxa across compartments: midgut and hindgut from third instar larvae of the Japanese beetle (*Popillia japonica* Newman), and associated soil. The natural log-ratio of taxa in each sample was calculated using qurro [68]. Taxa were chosen according to their prevalence among compartments (**Figure 3**) and their influence on variation along the first axis of the ordination space, Robust Aitchison PCA (**Figure 4**). Statistical significance (Brown-Forsythe Test) is indicated with one star (*) or two stars (**) for $p<0.05$ or $p<0.001$, respectively.

| **Comparison** | **Sample prevalence (%)** | | | **Location prevalence (%)** | | | **Brown-Forsythe Test** | | | ***p*-value for Posthoc Brown-Forsythe Test with Bonferroni correction** | | | | | |
|---|---|---|---|---|---|---|---|---|---|---|---|---|---|---|---|
| **Numerator/Denominator** | **Soil** | **Midgut** | **Hindgut** | **Soil** | **Midgut** | **Hindgut** | **Statistic** | ***p*-value** | | **Soil *vs.* Midgut** | | **Soil *vs.* Hindgut** | | **Midgut *vs.* Hindgut** | |
| *Bacillales/Bacteroidales* | 81 | 95 | 100 | 86 | 100 | 100 | 126.1 | 0.000 | ** | 0.278 | | 0.000 | ** | 0.000 | ** |
| *Bacillales/Betaproteobacteriales* | 100 | 90 | 100 | 100 | 100 | 100 | 39.5 | 0.000 | ** | 0.000 | ** | 0.357 | | 0.000 | ** |
| *Bacillales/Clostridiales* | 95 | 95 | 100 | 100 | 100 | 100 | 146.6 | 0.000 | ** | 0.754 | | 0.000 | ** | 0.000 | ** |
| *Bacillales/Desulfovibrionales* | 43 | 71 | 100 | 71 | 100 | 100 | 166.5 | 0.000 | ** | 0.187 | | 0.000 | ** | 0.000 | ** |
| *Bacteroidales/Betaproteobacteriales* | 81 | 86 | 100 | 86 | 100 | 100 | 63.4 | 0.000 | ** | 0.007 | * | 0.000 | ** | 0.000 | ** |
| *Bacteroidales/Desulfovibrionales* | 43 | 67 | 100 | 71 | 100 | 100 | 1.7 | 0.197 | | 1.000 | | 0.273 | | 0.482 | |
| *Chthoniobacterales/Bacillales* | 100 | 95 | 100 | 100 | 100 | 100 | 50.6 | 0.000 | ** | 0.000 | ** | 0.000 | ** | 1.000 | |
| *Chthoniobacterales/Bacteroidales* | 81 | 90 | 100 | 86 | 100 | 100 | 96.9 | 0.000 | ** | 0.002 | * | 0.000 | ** | 0.000 | ** |
| *Chthoniobacterales/Betaproteobacteriales* | 100 | 86 | 100 | 100 | 100 | 100 | 68.8 | 0.000 | ** | 0.048 | * | 0.000 | ** | 0.000 | ** |
| *Chthoniobacterales/Clostridiales* | 95 | 95 | 100 | 100 | 100 | 100 | 139.3 | 0.000 | ** | 0.000 | ** | 0.000 | ** | 0.000 | ** |
| *Chthoniobacterales/Desulfovibrionales* | 43 | 71 | 100 | 71 | 100 | 100 | 82.4 | 0.000 | ** | 0.019 | * | 0.000 | ** | 0.000 | ** |
| *Chthoniobacterales/Enterobacteriales* | 95 | 95 | 100 | 100 | 100 | 100 | 33.4 | 0.000 | ** | 0.000 | ** | 0.000 | ** | 0.711 | |
| *Clostridiales/Bacteroidales* | 81 | 90 | 100 | 86 | 100 | 100 | 0.9 | 0.400 | | 1.000 | | 0.122 | | 1.000 | |
| *Clostridiales/Betaproteobacteriales* | 95 | 86 | 100 | 100 | 100 | 100 | 88.6 | 0.000 | ** | 0.003 | * | 0.000 | ** | 0.000 | ** |
| *Clostridiales/Desulfovibrionales* | 43 | 71 | 100 | 71 | 100 | 100 | 0.5 | 0.586 | | 0.974 | | 0.608 | | 1.000 | |
| *Desulfovibrionales/Betaproteobacteriales* | 43 | 67 | 100 | 71 | 100 | 100 | 75.8 | 0.000 | ** | 0.026 | * | 0.000 | ** | 0.000 | ** |
| *Enterobacteriales/Bacillales* | 95 | 100 | 100 | 100 | 100 | 100 | 3.8 | 0.031 | * | 0.055 | | 0.031 | * | 1.000 | |
| *Enterobacteriales/Bacteroidales* | 81 | 95 | 100 | 86 | 100 | 100 | 75.4 | 0.000 | ** | 0.002 | * | 0.000 | ** | 0.000 | ** |

**Supplementary Table 10**. *Continued.*

| Comparison | Sample prevalence (%) | | | Location prevalence (%) | | | Brown-Forsythe Test | | | *p*-value for Posthoc Brown-Forsythe Test with Bonferroni correction | | | | | |
|---|---|---|---|---|---|---|---|---|---|---|---|---|---|---|---|
| **Numerator/Denominator** | **Soil** | **Midgut** | **Hindgut** | **Soil** | **Midgut** | **Hindgut** | **Statistic** | ***p*-value** | | **Soil *vs.* Midgut** | | **Soil *vs.* Hindgut** | | **Midgut *vs.* Hindgut** | |
| *Enterobacteriales/Betaproteobacteriales* | 95 | 90 | 100 | 100 | 100 | 100 | 21.5 | 0.000 | ** | 0.000 | ** | 0.021 | * | 0.002 | * |
| *Enterobacteriales/Clostridiales* | 90 | 95 | 100 | 100 | 100 | 100 | 72.1 | 0.000 | ** | 0.017 | * | 0.000 | ** | 0.000 | ** |
| *Enterobacteriales/Desulfovibrionales* | 43 | 71 | 100 | 71 | 100 | 100 | 90.0 | 0.000 | ** | 0.003 | * | 0.000 | ** | 0.000 | ** |
| *Rhizobiales/Bacillales* | 100 | 76 | 100 | 100 | 100 | 100 | 21.9 | 0.000 | ** | 0.012 | * | 0.000 | ** | 0.204 | |
| *Rhizobiales/Bacteroidales* | 81 | 71 | 100 | 86 | 100 | 100 | 89.5 | 0.000 | ** | 1.000 | | 0.000 | ** | 0.000 | ** |
| *Rhizobiales/Betaproteobacteriales* | 100 | 76 | 100 | 100 | 100 | 100 | 86.4 | 0.000 | ** | 0.006 | * | 0.000 | ** | 0.000 | ** |
| *Rhizobiales/Chthoniobacterales* | 100 | 76 | 100 | 100 | 100 | 100 | 12.9 | 0.000 | ** | 0.000 | ** | 0.006 | * | 0.063 | |
| *Rhizobiales/Clostridiales* | 95 | 76 | 100 | 100 | 100 | 100 | 124.9 | 0.000 | ** | 0.780 | | 0.000 | ** | 0.000 | ** |
| *Rhizobiales/Desulfovibrionales* | 43 | 57 | 100 | 71 | 100 | 100 | 114.1 | 0.000 | ** | 1.000 | | 0.000 | ** | 0.000 | ** |
| *Rhizobiales/Enterobacteriales* | 95 | 76 | 100 | 100 | 100 | 100 | 15.0 | 0.000 | ** | 0.018 | * | 0.000 | ** | 0.646 | |

**Supplementary Table 11.** Influence of location (all locations), soil management history (TPAC *versus* naturally infested locations), and manipulated larval treatment (Purdy *versus* Nursery) on variation in β-diversity of prokaryotic communities per compartment in the alimentary tract of third instar Japanese beetle (*Popillia japonica* Newman) larvae and associated soil. Permutational multivariate analysis of variance (PERMANOVA) and permutational analysis of multivariate dispersion (PERMDISP) methods were used to test differences between locations and homogeneity of dispersion among locations within each compartment. Test statistic (pseudo-F and F-value for PERMANOVA and PERMDISP, respectively) and *p*-values for both models using 999 permutations.

| | | | **Jaccard** | | **Bray-Curtis** | | **Unweighted UniFrac** | | **Weighted UniFrac** | | **DEICODE** | |
|---|---|---|---|---|---|---|---|---|---|---|---|---|
| | **Compartment** | **Method** | **Test statistic** | ***p*-value** | **Test statistic** | ***p*-value** | **Test statistic** | ***p*-value** | **Test statistic** | ***p*-value** | **Test statistic** | ***p*-value** |
| **All locations** Sample size=21 Groups=7 | Soil | PERMANOVA | 7.0 | 0.001 | 16.3 | 0.001 | 6.7 | 0.001 | 25.6 | 0.001 | 91.2 | 0.001 |
| | | PERMDISP | 0.4 | 0.323 | 0.5 | 0.249 | 0.4 | 0.350 | 0.6 | 0.203 | 0.3 | 0.700 |
| | Midgut | PERMANOVA | 2.1 | 0.001 | 3.4 | 0.001 | 2.2 | 0.001 | 2.3 | 0.008 | 8.9 | 0.001 |
| | | PERMDISP | 0.8 | 0.210 | 0.5 | 0.413 | 0.7 | 0.202 | 0.7 | 0.316 | 0.6 | 0.251 |
| | Hindgut | PERMANOVA | 1.6 | 0.001 | 1.8 | 0.001 | 2.0 | 0.001 | 2.6 | 0.005 | 4.9 | 0.001 |
| | | PERMDISP | 1.2 | 0.212 | 2.7 | 0.007 | 0.6 | 0.587 | 0.3 | 0.670 | 0.5 | 0.667 |
| **Soil management history** Sample size=18 Groups=2 | Soil | PERMANOVA | 3.1 | 0.003 | 3.6 | 0.006 | 3.0 | 0.007 | 2.3 | 0.083 | 11.8 | 0.005 |
| | | PERMDISP | 150.5 | 0.009 | 53.9 | 0.006 | 75.6 | 0.006 | 25.9 | 0.008 | 2.4 | 0.184 |
| | Midgut | PERMANOVA | 1.8 | 0.010 | 1.5 | 0.124 | 2.9 | 0.012 | 2.1 | 0.078 | 1.1 | 0.398 |
| | | PERMDISP | 4.1 | 0.653 | 2.3 | 0.184 | 1.0 | 0.457 | 0.0 | 0.909 | 4.5 | 0.082 |
| | Hindgut | PERMANOVA | 1.6 | 0.007 | 1.9 | 0.006 | 1.5 | 0.043 | 4.5 | 0.004 | 1.7 | 0.175 |
| | | PERMDISP | 13.5 | 0.086 | 44.2 | 0.009 | 6.6 | 0.061 | 3.3 | 0.124 | 2.1 | 0.183 |
| **Manipulated larval treatment** Sample size=6 Groups=2 | Soil | PERMANOVA | 6.4 | 0.099 | 13.5 | 0.100 | 6.9 | 0.114 | 21.9 | 0.104 | 3.9 | 0.102 |
| | | PERMDISP | 0.5 | 0.102 | 0.3 | 0.109 | 0.7 | 0.103 | 0.3 | 0.046 | 1.9 | 0.186 |
| | Midgut | PERMANOVA | 2.6 | 0.112 | 2.8 | 0.101 | 2.5 | 0.112 | 1.3 | 0.202 | 3.5 | 0.171 |
| | | PERMDISP | 5.7 | 0.107 | 26.4 | 0.043 | 2.9 | 0.086 | 10.6 | 0.121 | 0.1 | 0.808 |
| | Hindgut | PERMANOVA | 1.3 | 0.106 | 1.3 | 0.211 | 2.4 | 0.104 | 0.8 | 0.387 | 1.9 | 0.098 |
| | | PERMDISP | 6.2 | 0.087 | 1.9 | 0.104 | 0.8 | 0.045 | 0.0 | 0.897 | 11.1 | 0.372 |
